# Pheromone independent unisexual development in *Cryptococcus neoformans*

**DOI:** 10.1371/journal.pgen.1006772

**Published:** 2017-05-03

**Authors:** Rachana Gyawali, Youbao Zhao, Jianfeng Lin, Yumeng Fan, Xinping Xu, Srijana Upadhyay, Xiaorong Lin

**Affiliations:** Department of Biology, Texas A&M University, College Station, United States of America; Oregon State University, UNITED STATES

## Abstract

The fungus *Cryptococcus neoformans* can undergo **a**-α bisexual and unisexual reproduction. Completion of both sexual reproduction modes requires similar cellular differentiation processes and meiosis. Although bisexual reproduction generates equal number of **a** and α progeny and is far more efficient than unisexual reproduction under mating-inducing laboratory conditions, the α mating type dominates in nature. Population genetic studies suggest that unisexual reproduction by α isolates might have contributed to this sharply skewed distribution of the mating types. However, the predominance of the α mating type and the seemingly inefficient unisexual reproduction observed under laboratory conditions present a conundrum. Here, we discovered a previously unrecognized condition that promotes unisexual reproduction while suppressing bisexual reproduction. Pheromone is the principal stimulus for bisexual development in *Cryptococcus*. Interestingly, pheromone and other components of the pheromone pathway, including the key transcription factor Mat2, are not necessary but rather inhibitory for *Cryptococcus* to complete its unisexual cycle under this condition. The inactivation of the pheromone pathway promotes unisexual reproduction despite the essential role of this pathway in non-self-recognition during bisexual reproduction. Nonetheless, the requirement for the known filamentation regulator Znf2 and the expression of hyphal or basidium specific proteins remain the same for pheromone-dependent or independent sexual reproduction. Transcriptome analyses and an insertional mutagenesis screen in *mat2*Δ identified calcineurin being essential for this process. We further found that Znf2 and calcineurin work cooperatively in controlling unisexual development in this fungus. These findings indicate that Mat2 acts as a repressor of pheromone-independent unisexual development while serving as an activator for **a**-α bisexual development. The bi-functionality of Mat2 might have allowed it to act as a toggle switch for the mode of sexual development in this ubiquitous eukaryotic microbe.

## Author summary

Like many higher eukaryotes, the fungus *Cryptococcus neoformans* is known to undergo bisexual mating that involves α and **a** partners. Surprisingly, the α mating type is dominant in nature (>99%). Unisexual reproduction was previously discovered in *Cryptococcus* and the mating type α allele enhances this sexual reproduction mode. However, bisexual mating is far more robust than unisexual development under all current laboratory conditions tested. This challenges the predicted prevalence and importance of unisexual development in cryptococcal population. In this study, we identified a condition that favors unisexual reproduction and suppresses bisexual reproduction. We showed that unisexual reproduction occurring under this condition is independent of pheromone or the key components of the pheromone pathway, despite the fact that this pathway is crucial for bisexual development. Our findings demonstrate that the pheromone pathway is primarily used for identifying compatible mating partners through non-self recognition, and it is not required for pheromone-independent unisexual reproduction. This discovery opens the door to investigate how this eukaryotic microbe specifies different modes of sexual reproduction and the bifurcation of genetic control of these reproduction modes.

## Introduction

Sexual reproduction is generally considered bisexual, involving partners of the opposite sex. However, unisexual development (self-fertile or inbreeding) occurs in some eukaryotes without the need for an opposite mating partner [1–4]. Each reproduction mode has its own costs and benefits. Bisexual reproduction promotes outcrossing, but finding a compatible mating partner can be challenging. Unisexual reproduction promotes inbreeding/selfing, but it avoids the cost associated with locating a mating partner. The latter might be challenging for species that are immobile or species where compatible partners are rare.

Fungi are classified as homothallic or heterothallic based on their requirement for a compatible mating partner for sexual reproduction. The heterothallic life cycle or the **a**-α bisexual reproduction was elaborated for the fungus *Cryptococcus neoformans* in 1970s and it includes the yeast-to-hypha morphological transition and sporulation [5–7]. It was noticed that some isolates (mostly α) underwent a similar cellular differentiation process (filamentation and sporulation) on their own in the absence of cells of the opposite mating type. This process was termed “haploid fruiting” and was originally considered a mitotic or asexual event [8]. Later, “haploid fruiting” was found to be a meiotic process [9–11]. Sporulation during this process requires the key meiosis-specific genes and the resulting progeny show high levels of recombination frequencies [9, 11–14]. Such unisexual reproduction can occur by fusion between cells of the same mating type (the same sex mating) [9, 15], or more frequently, by endoreplication [16, 17].

**Fig 1:**
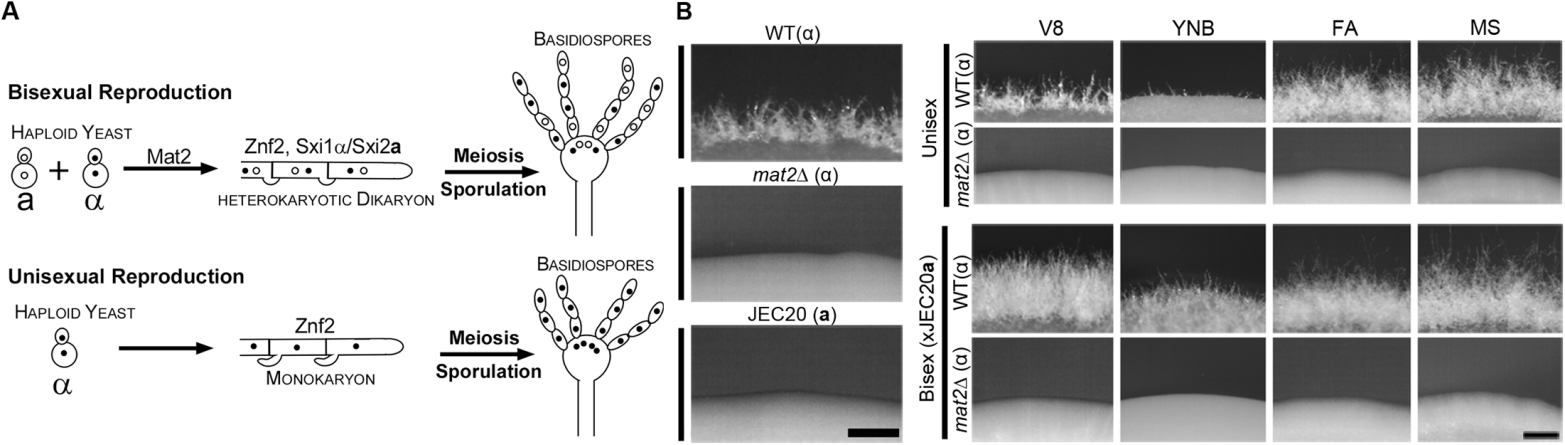
Mat2 is required for filamentation during bisexual and unisexual development under typical mating-inducing conditions. **(A)** Diagram depicting *Cryptococcus* bisexual (**a**-α) and unisexual (mostly α) reproduction. Both bisexual and unisexual reproduction culminate with the formation of fruiting structure basidium where meiosis and sporulation take place. The two sexual reproduction modes differ in the early developmental stages. Bisexual reproduction proceeds through **a**-α cell fusion for which Mat2 is essential. The Sxi1α and Sxi2**a** homeodomain complex is required for maintaining the **a**-α heterokaryotic stage. Unisexual reproduction can proceed either through cell fusion between two α cells or through endoduplication, and monokaryotic hyphae are formed. Znf2 is required for filamentation during both bisexual and unisexual reproduction. **(B)** The *mat2*Δ mutant is unable to undergo unisexual or bisexual reproduction under the known mating-inducing conditions. Equal number of wild-type (WT) and *mat2*Δ α cells were incubated alone on V8, YNB, FA, and MS medium in the dark at 22°C for 4 days (unisex, top right panel). JEC20**a** cells of equal number were mixed with either wild-type XL280 α or *mat2*Δ α cells. The co-cultures were incubated in the dark at 22°C for 4 days (bisex, bottom right panel). The mating partner JEC20**a** does not self-filament (left panel).

Both modes of sexual development in *Cryptococcus* involve the yeast-to-hypha morphological transition and the formation of fruiting bodies known as basidia and meiotic basidiospores (Fig 1A). Same environmental factors, such as nutrient limitation, dehydration, and copper, are known to influence unisexual and bisexual reproduction [18, 19]. Bisexual reproduction generates equal number of **a** and α progeny with comparable fitness [20–22], with the obvious exception that α isolates have enhanced ability to undergo unisexual development [9, 18, 21]. This offers a plausible explanation for the predominance of the α mating type (99%) among the clinical and environmental isolates [15, 16, 20, 23]. However, compared to bisexual reproduction, unisexual development is far less efficient in terms of robustness in filamentation (hyphal growth) and sporulation under laboratory conditions [9, 24, 25], presenting a challenge to the proposed importance of unisexual reproduction in nature.

Unisexual reproduction and bisexual reproduction have both shared and unique features (Fig 1A). However, no genetic factor that specifies cryptococcal commitment to unisexual development has been identified. Bisexual mating proceeds through cell fusion, the formation of dikaryotic hyphae, the fusion of two parental nuclei at basidium heads, and meiosis and sporulation. Unisexual development forms monokaryotic hyphae (or self-filamentation). Diploidization is highly plastic and can be derived from cell-cell fusion or endoreplication at any development stage prior to meiosis and sporulation at basidium heads [3, 26, 27]. Thus, the decision in choosing bisexual development must have occurred prior to cell fusion. The Sxi1α-Sxi2**a** homeodomain proteins are specific to bisexual reproduction, and they are critical in maintaining the dikaryotic state of the hyphae post α-**a** cell fusion [28, 29]. Because Sxi1α/Sxi2**a** are not necessary for α-**a-**conjugation [28, 29], this homeodomain complex functions after the choice of bisexual reproduction mode has been made. The master regulator of filamentation, Znf2, is required for filamentation and subsequent development to complete the life cycle during both unisexual and bisexual reproduction [30, 31] (Fig 1A). Thus, Znf2 is not specific to any reproduction mode. The transcription factor Mat2 activates the expression of multiple components of the pheromone sensing pathway by binding to the pheromone response elements (PREs) in the promoter region of the respective genes [30, 32]. Consequently, the *mat2*Δ mutant cannot undergo cell fusion even in the presence of a compatible wild-type mating partner [30]. We showed previously that Mat2 also plays an important role in deciding which parental mitochondrial DNA will be inherited in the progeny even prior to cell fusion (prezygotic control of uniparental mitochondria inheritance) [33]. Hence, Mat2 greatly influences the behavior of the mating partners prior to actual cell fusion, making it a likely candidate involved in early decision-making process for the mode of sexual development.

In sexual reproduction, meiosis (reduction division) follows polyploidization, which is achieved by **a**-α cell fusion during bisexual development. However, unisexual reproduction can proceed through cell fusion or endoreplication [3, 9]. We previously found that the frequency of cell fusion during unisexual mating was quite low [9] and evidence from population genetics studies indicates that endoreplication might be the major route for ploidy increase [16]. Thus it is conceivable that the pheromone pathway, which is designed for non-self-recognition between **a** and α cells but not for cells of the same mating type [34, 35], might not be as important for unisexual development. Some intriguing observations from previous studies support this notion [36–39]. For instance, deletion of the pheromone receptor gene *CPR*α (*STE3*) almost abolished bisexual mating (measured by quantifying **a**-α cell fusion events), but the abundance of hyphae produced by wild type and the *ste3*Δ mutant alone (self-filamentation) was similar [36]. Likewise, bisexual mating was impaired when the pheromone transporter gene *STE6* was disrupted, but the *STE6* gene deletion did not affect self-filamentation [38]. Similarly, when the G-protein α subunit genes *GPA2* and *GPA3* involved in sensing pheromone were both deleted, the *gpa2*Δ*gpa3*Δ mutant was sterile in bisexual mating but still robust in self-filamentation [39]. However, the finding that Mat2 was necessary for both unisexual and bisexual development [30, 31] is contradictory to the notion that the pheromone pathway might not be critical for unisexual development. We set out this study to resolve the conflict and to investigate if one of the roles of the pheromone pathway is priming *Cryptococcus* to choose bisexual over unisexual reproduction. Our results indicate that unisexual reproduction can be completed independent of the pheromone pathway under multiple disparate conditions, thus resolving previously contradictory findings.

## Results

### The *mat2*Δ mutant is able to undergo self-filamentation under mating inducing conditions

Mat2 regulates the pheromone response pathway and is required for cell fusion during bisexual (**a**-α) mating [30–32]. The failure in **a**-α cell fusion blocks further development into dikaryotic hyphae (Fig 1A). When non-self-filamentous strains are used, successful **a**-α cell fusion during bisexual mating will lead to production of dikaryotic hyphae (Fig 1B). As expected, no hypha differentiation was observed when the *mat2*Δ mutant was co-cultured with an opposite mating partner under all mating-inducing conditions tested (bottom panel, Fig 1B), consistent with the established role of Mat2 in the pheromone pathway. XL280 is a self-filamentous strain commonly used to study unisexual development [10, 12–14, 18]. The deletion of *MAT2* in XL280 abolished self-filamentation under all mating-inducing conditions tested (V8, Yeast Nitrogen Base, Filament Agar, and Murashige and Skoog media) (top panel, Fig 1B) [30]. Thus, Mat2 was considered crucial for both unisexual and bisexual reproduction.

**Fig 2:**
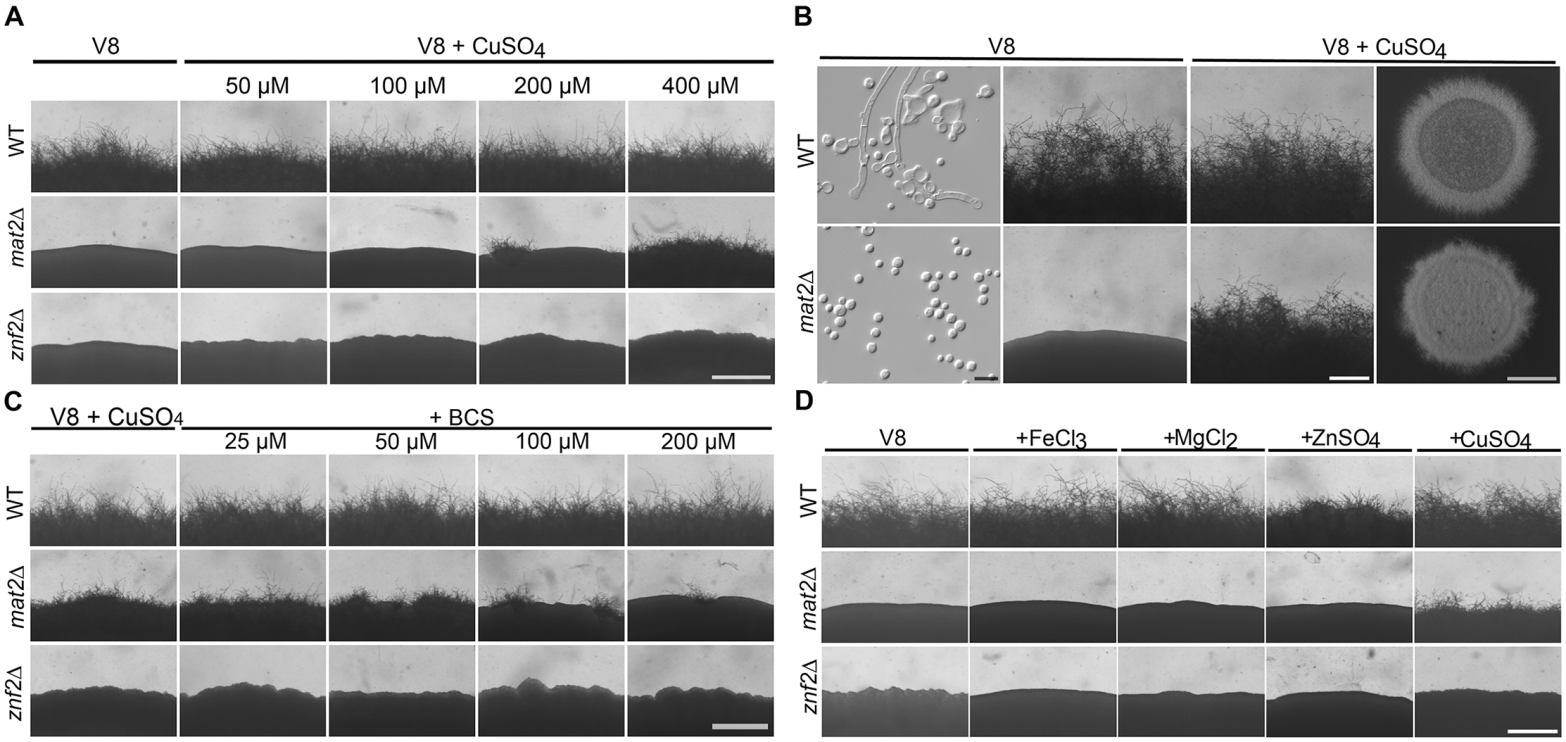
The *mat2*Δ mutant can filament on V8+copper medium. **(A)** The wild type (WT), *mat2*Δ, and *znf2*Δ strains were cultured on V8 medium or V8 medium supplemented with different concentrations of copper for a week. **(B)** Colony and cellular morphology of WT and the *mat2*Δ mutant cultured on V8 or V8+copper (400 μM) medium. Scale bar for cellular morphology examination on the left column is 10 μm. Scale bars for column 3 and 4 are 500 μm and 2000 μm respectively. **(C)** WT, *mat2*Δ, and *znf2*Δ were cultured on V8+copper (400 μM) medium with different concentrations of the copper chelator BCS. Scale bar: 500 μm. **(D)** WT, *mat2*Δ, and *znf2*Δ were cultured on V8 medium supplemented with 400 μM of the tested metal ions under the same conditions as in panel A. Scale bar: 500 μm.

If the hypothesis of the non-essentiality of the pheromone pathway for unisexual development is valid, then we would predict that the disruption of Mat2, the master regulator of the pheromone sensing pathway, should not abolish the ability of *Cryptococcus* to undergo self-filamentation. We previously found that copper (≤ 100 μM) enhances self-filamentation [18]. Copper is also one of the components in the V8 juice medium that promote mating [19]. Thus, we decided to test the impact of copper at various concentrations on the *mat2*Δ mutant. The colony of the *mat2*Δ mutant grown on V8 medium was smooth and round due to the presence of only yeast cells (Fig 2A and 2B), as expected based on previous studies [30, 32]. We found that copper at 400 μM induced self-filamentation in the *mat2*Δ mutant (Fig 2A). Accordingly, the colony of the *mat2*Δ mutant grown on the V8+copper medium appeared fluffy due to profuse production of filaments (Fig 2B). Consistently, the addition of the copper chelator BCS (bathocuproinedisulfonic acid) to the V8+copper medium (400 μM) reduced the robustness of filamentation shown by the *mat2*Δ mutant in a dose-dependent manner (Fig 2C). None of the other metal ions tested, namely iron, magnesium or zinc, induced filamentation in the *mat2*Δ mutant (Fig 2D). Addition of copper to defined media such as Filament Agar medium or Yeast Nitrogen Base medium also triggered filamentation in the *mat2*Δ mutant (S1 Fig), indicating that the effect of copper on Mat2-independent filamentation is not limited to the complex V8 juice medium.

To examine if the ability of the *mat2*Δ mutant to filament in response to copper is specific to the XL280 background, we tested the *mat2*Δ mutant made in the JEC21 background. Wild type JEC21 produced sporadic filaments on V8 medium and filamentation was slightly increased on V8+copper medium (S2 Fig). The *mat2*Δ mutant in the JEC21 background filamented more robustly on V8+copper medium (S2 Fig). The *mat2*Δ mutant was also able to produce basidia and spores on V8+copper medium, although at a lower frequency (S3 Fig). We also tested the *mat2*Δ mutants made in the backgrounds of two congenic pairs: XL280α/XL280**a** (S1 Fig) and JEC21α/JEC20**a** (S4 Fig). The *mat2*Δ mutants, either of the mating type α or the mating type **a**, filamented on V8+copper and FA+copper media. Taken together, the *mat2*Δ mutant made in different genetic or mating type backgrounds can undergo self-filamentation and sporulation in response to copper. Thus, unisexual reproduction does not depend on this transcription factor of the pheromone pathway.

### Filamentation shown by the *mat2*Δ mutant requires Znf2

Mat2 controls multiple components of the pheromone sensing pathway and deletion of the *MAT2* gene abolishes the ability of cells to produce or to respond to pheromone [30, 32]. Under mating-inducing condition, Mat2 activates Znf2, the master regulator of filamentation [30, 31]. However, Znf2 itself is not critical for the pheromone sensing or response [30].

**Fig 3:**
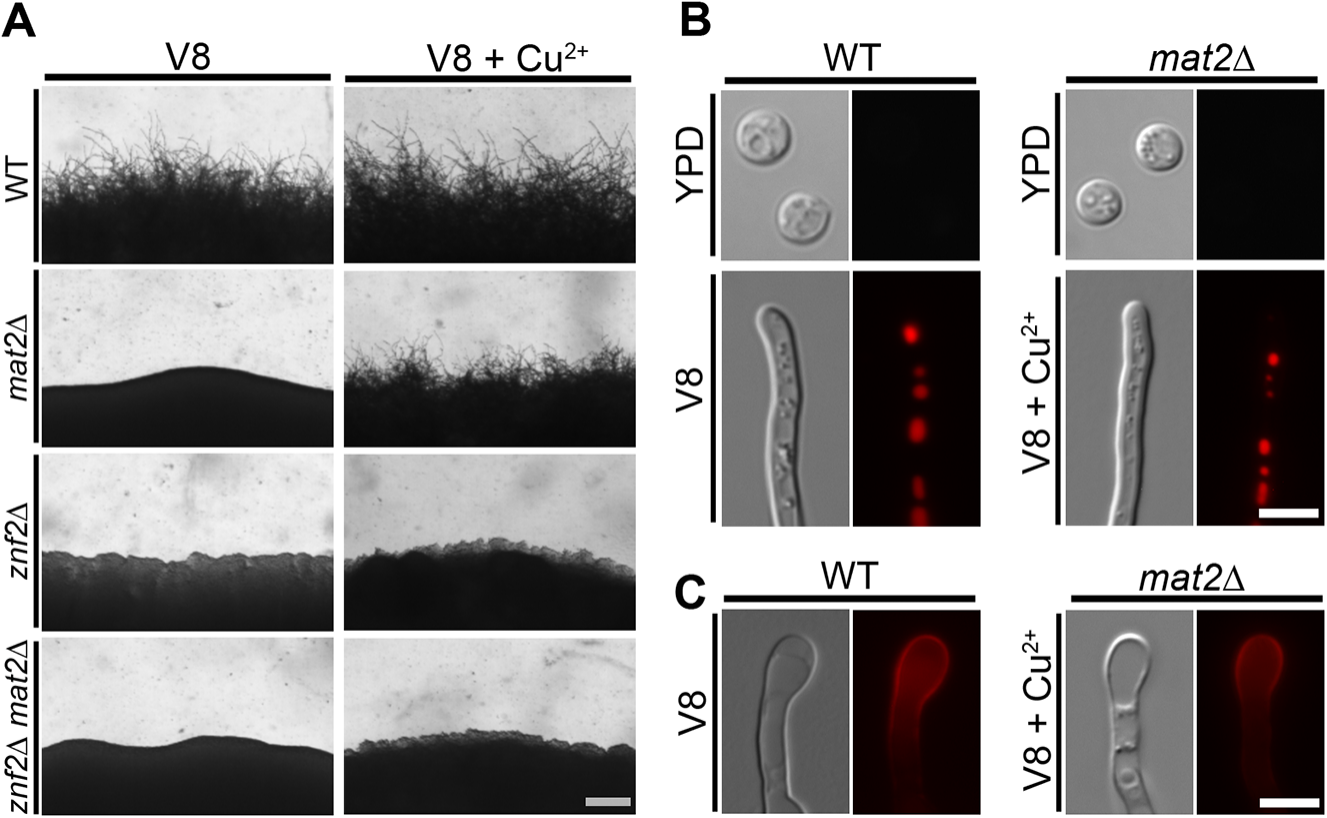
Filamentation shown by the *mat2*Δ mutant requires the morphogenesis regulator Znf2. **(A)** Wild type, *mat2***Δ**, *znf2***Δ**, and *znf2***Δ***mat2***Δ** strains were cultured on V8 or V8+copper medium in the dark at 22°C for 7 days before the pictures were taken. Scale bar: 200 μm. **(B)** Wild type and *mat2***Δ** expressing Cfl1-mCherry under its native promoter were cultured on YPD medium, V8 medium, or V8+Cu^2+^ medium. **(C)** Wild type and *mat2***Δ** expressing P_*GPD1*_-Dha1-mCherry were cultured on V8 or on V8+Cu^2+^ medium. Scale bars: 5 μm.

To determine if filamentation in the *mat2*Δ mutant evoked by copper still requires Znf2, we generated the *mat2*Δ*znf2*Δ double mutant. Although the *znf2*Δ mutant does not self-filament, the *znf2*Δ mutant can mate with a wild-type partner of a compatible mating type during bisexual mating on V8 medium and the fused heterokaryon will produce filaments (S5 Fig) [30]. The *mat2*Δ*znf2*Δ double mutant, however, failed to filament when co-cultured with the compatible wild-type partner on V8 medium (S5 Fig), consistent with the essential role of Mat2 in cell fusion. We then tested self-filamentation of this *znf2*Δ*mat2*Δ double mutant on V8+copper medium. As expected, the *mat2*Δ mutant filamented on V8+copper medium. The *znf2*Δ*mat2*Δ double mutant failed to filament on V8+copper medium, similar to the *znf2*Δ single mutant (Fig 3A). These results indicate that filamentation in the *mat2*Δ mutant stimulated by copper still requires Znf2. The finding further corroborates Znf2 as the essential regulator of filamentation.

To examine if filaments produced by the *mat2***Δ** mutant on V8+copper share the same molecular features as filaments produced by the wild type, we examined the expression pattern of a few proteins known to be expressed in wild-type hyphae. We first examined the hypha-specific protein Cfl1 [31]. Cfl1-mCherry expressed under the control of *CFL1*’s native promoter was detected in hyphal subpopulations when wild-type cells were cultured on V8 medium, but not in yeast cells when the wild-type strain was cultured in YPD medium (Fig 3B). As expected, no Cfl1-mCherry could be detected when the *mat2***Δ** mutant was cultured on YPD or on V8 medium (Fig 3B), consistent with the *mat2***Δ** mutant forming only yeast cells under these conditions. By contrast, Cfl1 could be clearly detected on the hyphae produced by the *mat2***Δ** mutant grown on V8+copper medium (Fig 3B). Another protein, Dha1, which is enriched in basidia produced by wild-type hyphae [40], showed similar localization in the basidia produced by either the wild type cultured on V8 or the *mat2***Δ** mutant cultured on V8+copper medium (Fig 3C). These results suggest that filaments produced by the *mat2***Δ** mutant shares the same molecular features with that of the wild type.

### Filamentation in the *mat2*Δ mutant is independent of the pheromone sensing pathway

**Fig 4:**
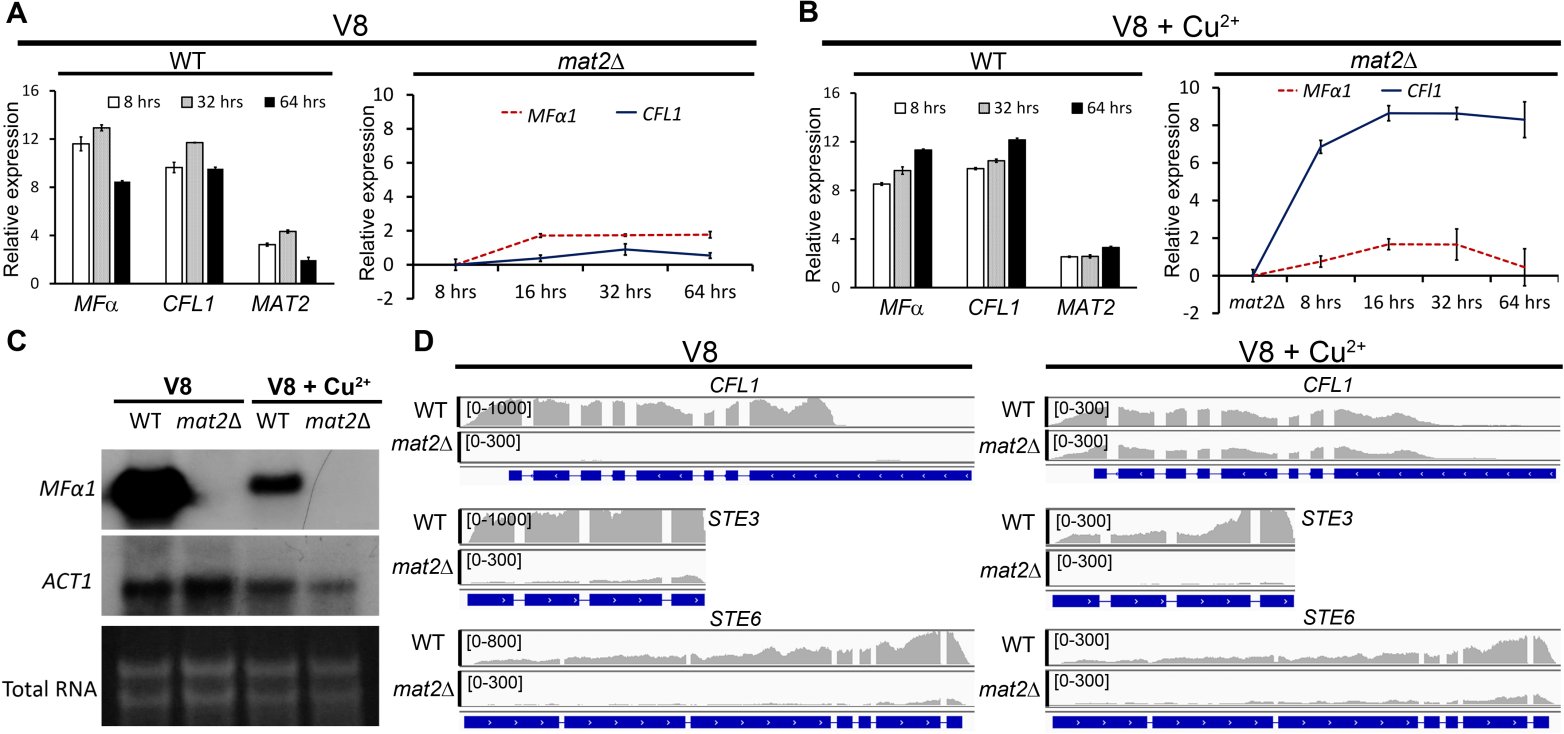
Filamentation of the *mat2*Δ mutant elicited by copper is independent of the pheromone pathway. **(A)** The relative transcript levels of the pheromone gene *MFα*, *CFL1*, and *MAT2* in WT and the *mat2*Δ mutant cultured on V8 medium at the indicated time points measured by RT-PCR. **(B)** The relative transcript level of *MFα*, *CFL1*, and *MAT2* in WT and *mat2*Δ mutant cultured on V8+Cu^2+^ medium at the indicated time points measured by RT-PCR. **(C)** Northern blot probed for the *MF*α transcripts in WT and the *mat2*Δ mutant cultured on either V8 or V8+Cu^2+^ medium for 16 hours. The *ACT1* transcripts and also the rRNAs were used as loading controls. **(D)** FPKMs (Fragments Per Kilobase of exon per Million fragments mapped) of the filamentation marker *CFL1*, the pheromone receptor gene *STE3*, and the pheromone transporter gene *STE6* in WT and the *mat2*Δ mutant cultured on V8 or V8+Cu^2+^ medium for 16 hours based on the RNA-seq data. The scales for reads count were set to different threshold values for easy visualization.

The ability of the *mat2***Δ** mutant to filament on V8+copper medium could be caused by cryptic activation of pheromone. To test this, we examined the transcript level of the pheromone gene *MFα1* in wild type and in *mat2*Δ cultured on V8 or V8+copper medium for the indicated time periods. *CFL1*, the hyphal specific gene downstream of Znf2 was used as a marker for filamentation [31]. As expected, the *CFL1* transcript level was increased when wild type was cultured on V8 medium compared to that on YPD medium (Fig 4A). The *MFα1* transcript level was also induced in wild type cultured on V8 medium (Fig 4A), consistent with the known induction of pheromone under this condition [18, 30]. By contrast, there was no induction in the *CFL1* transcript level or the *MFα1* transcript level in the *mat2*Δ mutant on V8 medium (Fig 4A), which corroborates the established role of Mat2 in the pheromone pathway and the non-filamentous phenotype of the *mat2*Δ mutant under this condition. Consistent with the real-time PCR results, northern blot analysis also indicated no induction for *MFα1* in the *mat2*Δ mutant cultured on V8 medium (Fig 4C). On V8+copper medium, the transcript level for both *CFL1* and *MFα1* were increased in wild type compared to that in YPD medium (Fig 4B). For the *mat2*Δ mutant cultured on V8+copper medium, the transcript level of *CFL1* was increased (Fig 4B). This is consistent with the expression of Cfl1 protein (Fig 3B) and the filamentous phenotype of the *mat2*Δ mutant cultured under this condition. However, there was no induction of *MFα1*. Similarly, no *MFα1* was detected in the *mat2*Δ mutant on V8+copper medium by northern blot (Fig 4C). Thus, regardless of the conditions used, there was no detectable induction of pheromone in the *mat2***Δ** mutant.

To further attest pheromone-independent nature of filamentation in the *mat2*Δ mutant, we compared the transcript level of other components of the pheromone sensing pathway in the wild type and the *mat2*Δ mutant on V8 and V8+copper medium using RNA-seq. Regardless of the condition used, we found that the transcript levels for many components in the pheromone pathway were low in the *mat2*Δ mutant, including the pheromone transporter gene *STE6* and the pheromone receptor gene *STE3* (Fig 4D). By contrast, the transcript level of *CFL1* was induced in the *mat2*Δ mutant on V8+copper medium, consistent with the filamentous phenotype of the *mat2*Δ mutant under this condition. Phenotypic examination of the triple pheromone mutant *mfα1–3*Δ and the pheromone transporter *ste6*Δ mutant revealed slightly enhanced self-filamentation of these mutants when cultured on V8+copper medium (S6 Fig). Collectively, these results demonstrate that filamentation in *mat2*Δ on V8+copper medium is independent of pheromone or the components of the pheromone sensing pathway.

**Fig 5:**
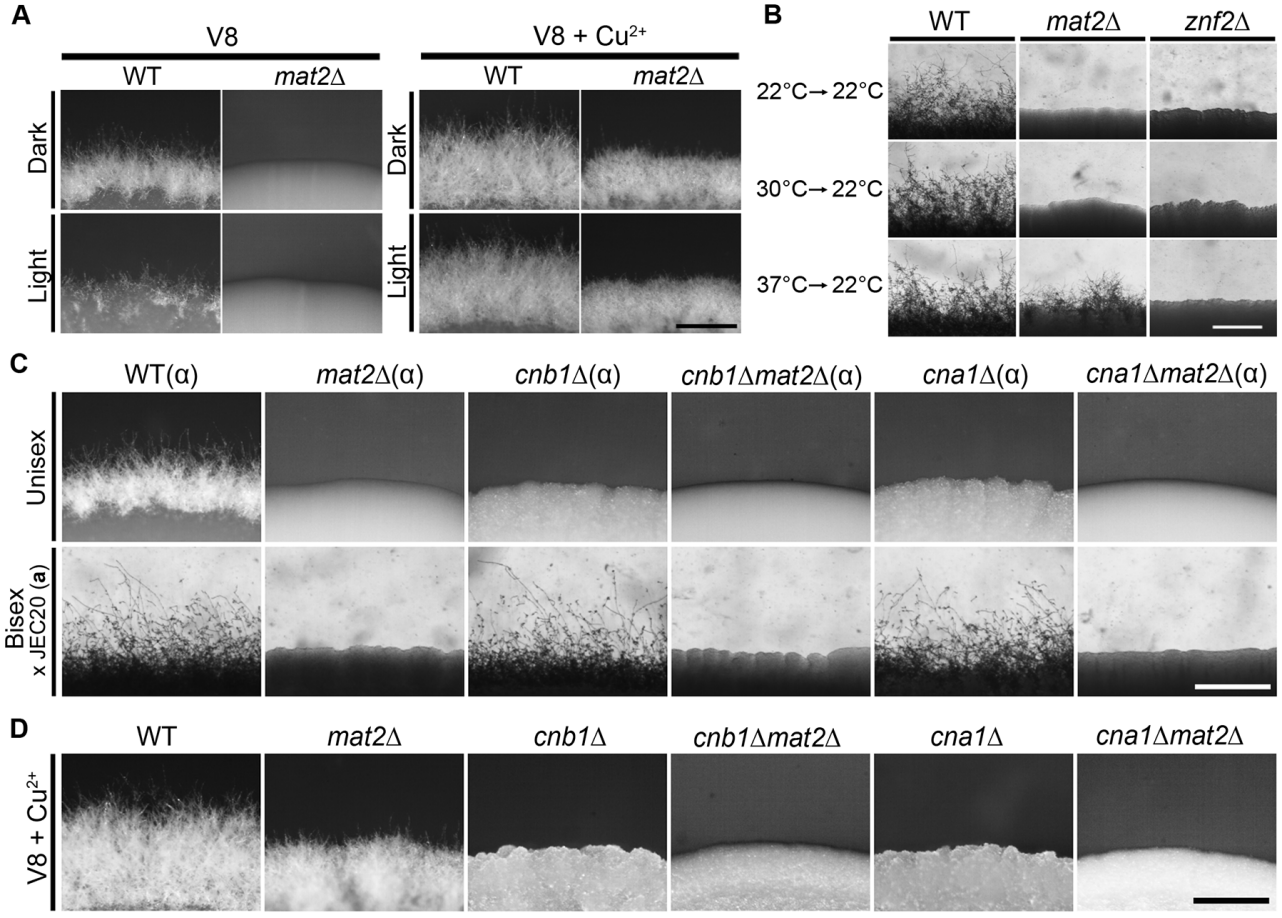
Influence on filamentation in the *mat2*Δ mutant by light, prior exposure to high temperature, and calcineurin. **(A)** WT and *mat2*Δ were cultured on V8 or V8+Cu^2+^ medium and incubated at 22°C in the dark or under constant light. **(B)** WT, *mat2*Δ, and *znf2*Δ were grown on YPD at 37°C, 30°C, or 22°C for 36 hours. Cells were transferred to V8 medium and incubated in the dark at 22°C for 8 days before the pictures were taken. **(C)** WT, *mat2*Δ, *cnb1*Δ, *cnb1*Δ*mat2*Δ, *cna1*Δ, *cna1*Δ*mat2*Δ of the α mating type were cultured on V8 medium either alone (unisex) or mixed with JEC20a (bisex) for 6 days. (**D**) The same strains as in panel C were cultured alone on V8+Cu^2+^ medium for 6 days. Scale bars: 500 μm.

As the *mat2*Δ mutant can filament independent of the pheromone pathway on V8+copper medium, one would predict that factors affecting pheromone production should not affect *mat2*Δ filamentation. Light is known to repress the pheromone genes through the light sensor complex [41]. Here, we cultured the wild type and the *mat2*Δ mutant strains on V8 or V8+copper medium in the dark or under constant light. Wild type cultured on V8 medium showed drastic reduction in filamentation when it was exposed to constant light (Fig 5A), consistent with the idea that filamentation on V8 medium is primarily driven by the pheromone pathway. The *mat2*Δ mutant grown on V8 medium did not show any filamentation either under constant light or in the dark. Both wild type and the *mat2*Δ mutant filamented equally well on V8+copper medium in the dark or in presence of light (Fig 5A). These results indicate that light does not inhibit filamentation on V8+copper medium, consistent with our prediction of its pheromone independent nature.

A previous study showed that prior growth at a high temperature can prime self-filamentation in *C*. *neoformans* once cells are transferred to filamentation-inducing conditions [42]. We decided to test if prior growth at a high temperature can induce filamentation in the *mat2*Δ mutant. We cultured the wild type, the *mat2*Δ mutant, and the *znf2*Δ mutant at 22°C, 30°C, and 37°C on YPD medium before transferring them onto V8 medium for additional incubation at 22°C. As expected, wild type filamented and the *znf2*Δ mutant showed no filamentation under all three conditions tested (Fig 5B). The *mat2*Δ mutant transferred from prior cultures at 37°C, but not at 22°C or 30°C, filamented (Fig 5B). This finding indicates that high temperature-induced filamentation can be independent of pheromone.

### Suppressor screen to identify factors critical for *mat2*Δ filamentation

To identify components required for filamentation in the *mat2*Δ mutant, we decided to perform forward genetic screen in the *mat2*Δ mutant background via *Agrobacterium*-mediated insertional mutagenesis. We generated 77,000 T-DNA insertional mutants in the *mat2*Δ background and screened these mutants on V8+copper medium for isolates that failed to filament (S7A Fig). We isolated 47 insertional mutants that showed only yeast growth on V8+copper medium. Genomic DNA of these selected insertional mutants were pooled into four groups and sequenced, and the T-DNA insertion sites were identified using a recently described approach [43]. A total of 93 insertion sites were identified (S7B Fig), indicating that multiple insertions occurred in some strains and that not all insertions were responsible for blocking filamentation. Indeed, T-DNA insertions into CNB05100 and CNC04530 were found to be not responsible for abolishing filamentation on copper medium based on our independent gene deletion experiments. Of the identified insertions, 32 occurred in intergenic regions and the rest inserted within ORFs. A couple of insertions occurred in the same regions. Genetic loci affected by the T-DNA insertions from the 47 mutants and their annotated gene functions are included in S3 Table. These genes encode membrane proteins, mitochondrial proteins, transporters, and hypothetic proteins. Some of the insertion sites were also identified through inverse PCR as previously described [30]. Because the *mat2*Δ parental strain is sterile in bisexual mating, we could not perform genetic linkage analysis by crossing to separate the insertions that are linked to the non-filamentous phenotype from those that are unlinked. By combining the T-DNA insertional mutagenesis data and the RNA-seq data, we selected a few candidate genes of interest for targeted gene deletion. The deletion of the genes recapitulated the phenotype caused by the insertions.

### Calcineurin is required for pheromone-dependent and pheromone-independent filamentation

A total of 1068 genes were upregulated by more than 2 fold in the *mat2*Δ mutant cultured on V8+copper (filamentous colony) compared to that on V8 (yeast colony) based on our RNA-seq data (S4 Table). To narrow down the genes that are unique for filamentation in the *mat2*Δ mutant, we compared transcripts of the *mat2*Δ mutant and wild type grown on V8+copper to those grown on V8 that were upregulated for more than 3 fold. A total of 196 transcripts were shared between wild type and the *mat2*Δ mutant that were upregulated on V8+copper and 264 transcripts were unique to the *mat2*Δ mutant (S8A Fig). We found that multiple components of the calcineurin pathway were upregulated in the *mat2*Δ mutant on V8+copper medium (S8B Fig). One of the candidate genes identified through DNA sequencing as well as through inverse PCR sequencing of the insertional mutants was *CNB1*. *CNB1* encodes the regulatory subunit of calcineurin, a serine threonine specific phosphatase [44–46]. Calcineurin is a heterodimer composed of the catalytic subunit Cna1 and the regulatory subunit Cnb1. Calcineurin is required for fungal adaptation to different environment conditions such as ion stress, pheromone response, morphogenesis, and growth at 37°C [45].

To confirm that the disruption of *CNB1* abolished filamentation, we deleted the *CNB1* gene in the wild-type XL280 background. The deletion of *CNB1* caused severe growth defect at 37°C (S9 Fig), consistent with what was reported previously of the *cnb1*Δ mutant made in the JEC21 background [44]. The *cnb1*Δ strain failed to produce any filament either on V8 medium or V8+copper medium (Fig 5C and 5D). This observation suggests that the Cnb1 is required for filamentation regardless of the pheromone response. To test if the pheromone response pathway is functional in the *cnb1*Δ mutant made in the XL280 background, we crossed the *cnb1*Δα cells with JEC20 (**a**). Because JEC20 is a mating type **a** strain that does not self-filament, any filaments observed from the cross would be the result of cell fusion events between *cnb1*Δ α mutant and JEC20 (**a**). Filamentation was observed from the cross between *cnb1*Δ α and JEC20 (**a**) (Fig 5C), indicating that Cnb1 is not critical for the pheromone response. This is again consistent with previous studies in the JEC21 background, indicating that the deletion of *CNB1* does not have any defect in pheromone production or cell fusion [47].

To further confirm that the deletion of *CNB1* is responsible for the blocked filamentation of *mat2*Δ on V8+copper medium identified by our insertional mutagenesis screen, we generated the *cnb1*Δ*mat2*Δ double mutant. The *cnb1*Δ*mat2*Δ double mutant did not produce any filaments when crossing with JEC20 (**a**) (Fig 5C), consistent with the cell fusion defect caused by the deletion of *MAT2* [30]. The *cnb1*Δ*mat2*Δ double mutant failed to undergo self-filamentation either on V8 or V8+copper medium (Fig 5C and 5D), supporting the idea that Cnb1 is required for both pheromone-dependent and pheromone-independent filamentation. Similar to the deletion of *CNB1*, the deletion of the catalytic subunit of calcineurin *CNA1* did not prevent crossing with a compatible wild-type mating partner during bisexual mating, but it blocked self-filamentation (Fig 5C). The double mutant *cna1*Δ*mat2*Δ, similar to the double mutant *cnb1*Δ*mat2*Δ, was unable to filament either on V8 or V8+copper medium (Fig 5C and 5D).

**Fig 6:**
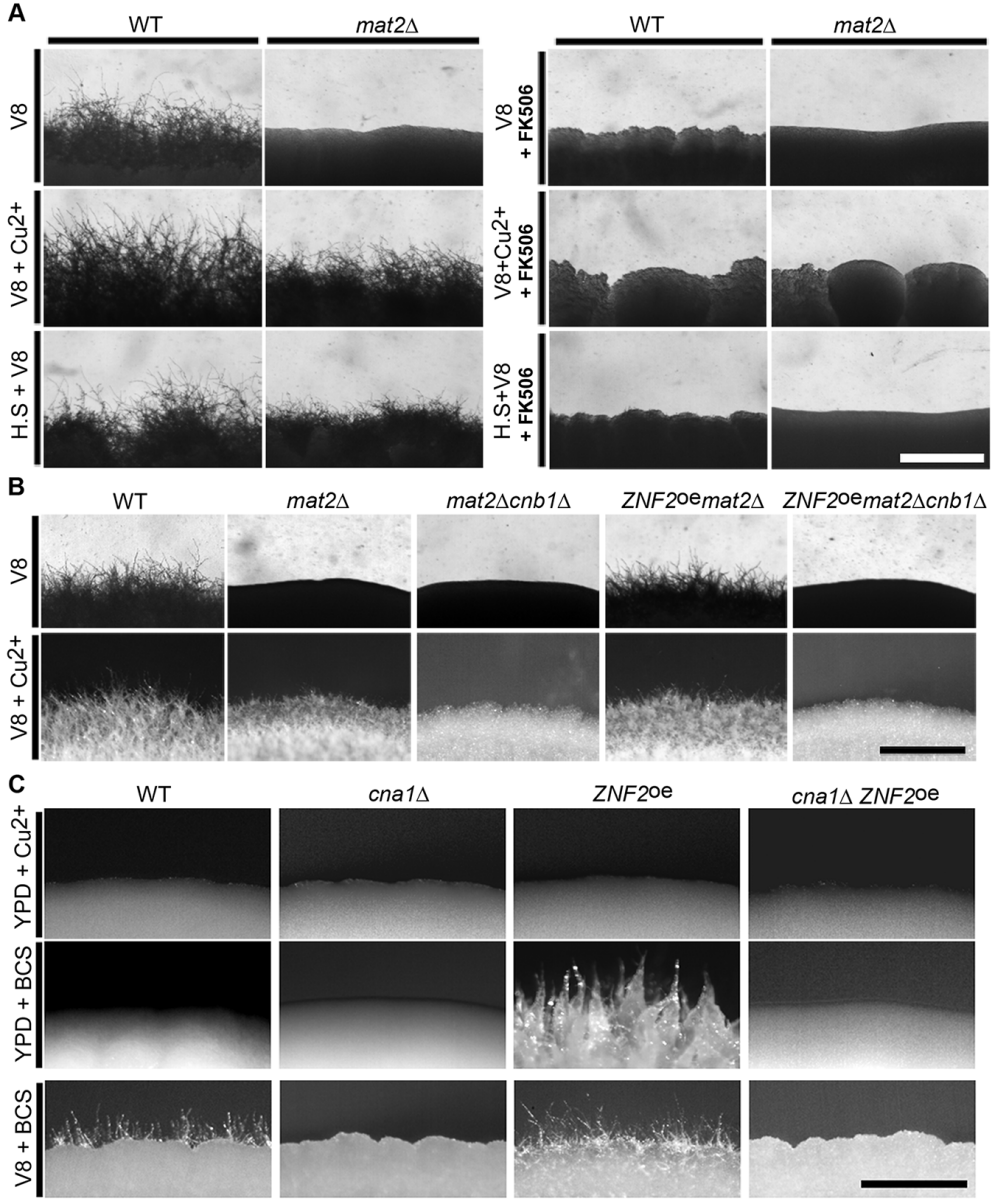
Filamentation requires both Znf2 and calcineurin. **(A)** WT and *mat2*Δ were incubated on V8 medium, V8+copper medium, or V8 medium after prior exposure to high temperature in the absence or the presence of the calcineurin inhibitor FK506. Pictures were taken after 1 week of incubation in the dark at 22°C. **(B)** WT, *mat2*Δ, *mat2*Δ*cnb1*Δ, P_*GPD1*_-*ZNF2 mat2*Δ, and P_*GPD1*_-*ZNF2 mat2*Δ*cnb1*Δ strains were cultured on V8 or V8+Cu^2+^ medium. **(C)** WT, *cna1*Δ, P_*CTR4*_-*ZNF2*, and *cna1*Δ P_*CTR4*_-*ZNF2* strains were cultured on *ZNF2*-suppressing YPD+Cu^2+^ medium, and *ZNF2*-inducing YPD+BCS or V8+BCS medium. Scale bars: 500 μm.

Since calcineurin is activated in response to increased calcium levels [48], we examined if addition of CaCl_2_ could induce filamentation in the *mat2*Δ mutant. Indeed, we found that addition of CaCl_2_ at high concentrations (between 1000–2000 μM) induced filamentation in the *mat2*Δ mutant after prolonged incubation in the dark on V8 medium (S8C Fig). We showed earlier that prior exposure to high temperature also induced filamentation in the *mat2*Δ mutant. Given that calcineurin is activated in response to calcium and also to environmental stresses such as high temperature [49, 50], we reasoned that filamentation induced by high temperature may also require calcineurin. We used the calcineurin inhibitor FK506 to test this hypothesis as the deletion of the *CNA1* gene or the *CNB1* gene causes severe growth defect at 37°C (S9 Fig) [47]. Here, we cultured wild type and the *mat2*Δ mutant at 37°C and then transferred the cells onto V8 medium containing FK506 (1 μg/ml). No filamentation was observed in either wild type or the *mat2*Δ mutant (Fig 6A), indicating that treatment with FK506 abolished filamentation. Hence, thermo-induced filamentation also requires calcineurin. Collectively, these data confirm that filamentation, be it pheromone-dependent or pheromone-independent, requires calcineurin.

Because Crz1 is a known transcription factor downstream of calcineurin and because its transcript level was induced in the *mat2*Δ mutant on V8+copper medium (S8B Fig), we decided to examine if calcineurin acts on copper-induced filamentation through Crz1. For this purpose, we generated the *crz1*Δ mutant as well as the *crz1*Δ*mat2*Δ double mutant. Either the *crz1*Δ mutant or the *crz1*Δ*mat2*Δ double mutant showed obvious growth defect at 37°C (S9 Fig), indicating that Crz1 is not as critical as calcineurin for thermal-adaptation. Such phenotype is expected based on the *crz1*Δ mutant phenotype reported for the serotype A background [49]. However, the deletion of *CRZ1* in the wild-type background did not affect filamentation on V8 or V8+copper medium. Likewise, the deletion of *CRZ1* in the *mat2*Δ mutant background did not affect the ability of *mat2*Δ to filament on V8+copper medium (Fig 6D). This result suggests that Crz1 is dispensable for filamentation on copper medium. Thus, downstream factors of the calcineurin pathway other than Crz1 [49] must be required for filamentation induced by copper.

### Znf2 and calcineurin cooperate in regulating filamentation

Znf2 is a specific regulator for hyphal morphogenesis and it is not critical for adaptation to many other stresses tested [30, 31]. Consistently, no enrichment of stress regulatory elements (STEs) was observed within 2 kb sequences upstream of the open reading frame of *ZNF2*. Although the core sequence of metal regulatory elements (5’-GCTG-3’) [51] are enriched in this region, the copper specific sensing element (5’-ATATTGCTGT-3’) is absent. This morphogenesis regulator is expressed at low levels except under conditions that induce filamentation. So far no factor has been identified that can override the need for Znf2 in terms of filamentation. It is thus not surprising that overexpression of *ZNF2* can enable filamentation in various mutants (e.g. *mat2*Δ). Like Znf2, calcineurin is also essential for Mat2-dependent or Mat2-independent filamentation (this study and previous studies [30, 47]). In contrast to Znf2, calcineurin is a general stress response regulator in *C*. *neoformans* and in other fungi [52–56]. We hypothesize that this general stress adaptation regulator cooperates with Znf2 in controlling filamentation in *Cryptococcus*. If this hypothesis is valid, then the defect in filamentation caused by the disruption of calcineurin would not be overcome by overexpressing *ZNF2*. To test this hypothesis, we introduced the *ZNF2* overexpression construct into the *mat2*Δ and the *mat2*Δ*cnb1*Δ double mutant. Overexpression of *ZNF2* in the *mat2*Δ mutant enabled filamentation (Fig 6B), as we reported previously [31]. However, overexpression of *ZNF2* in the *mat2*Δ*cnb1*Δ mutant failed to confer filamentation (Fig 6B). Consistently, deletion of the *CNA1* gene abolished filamentation in the *ZNF2*^oe^ strain on V8 medium or V8+copper medium (Fig 6C). These results indicate that both Znf2 and calcineurin are required for pheromone-dependent and pheromone-independent filamentation.

**Fig 7:**
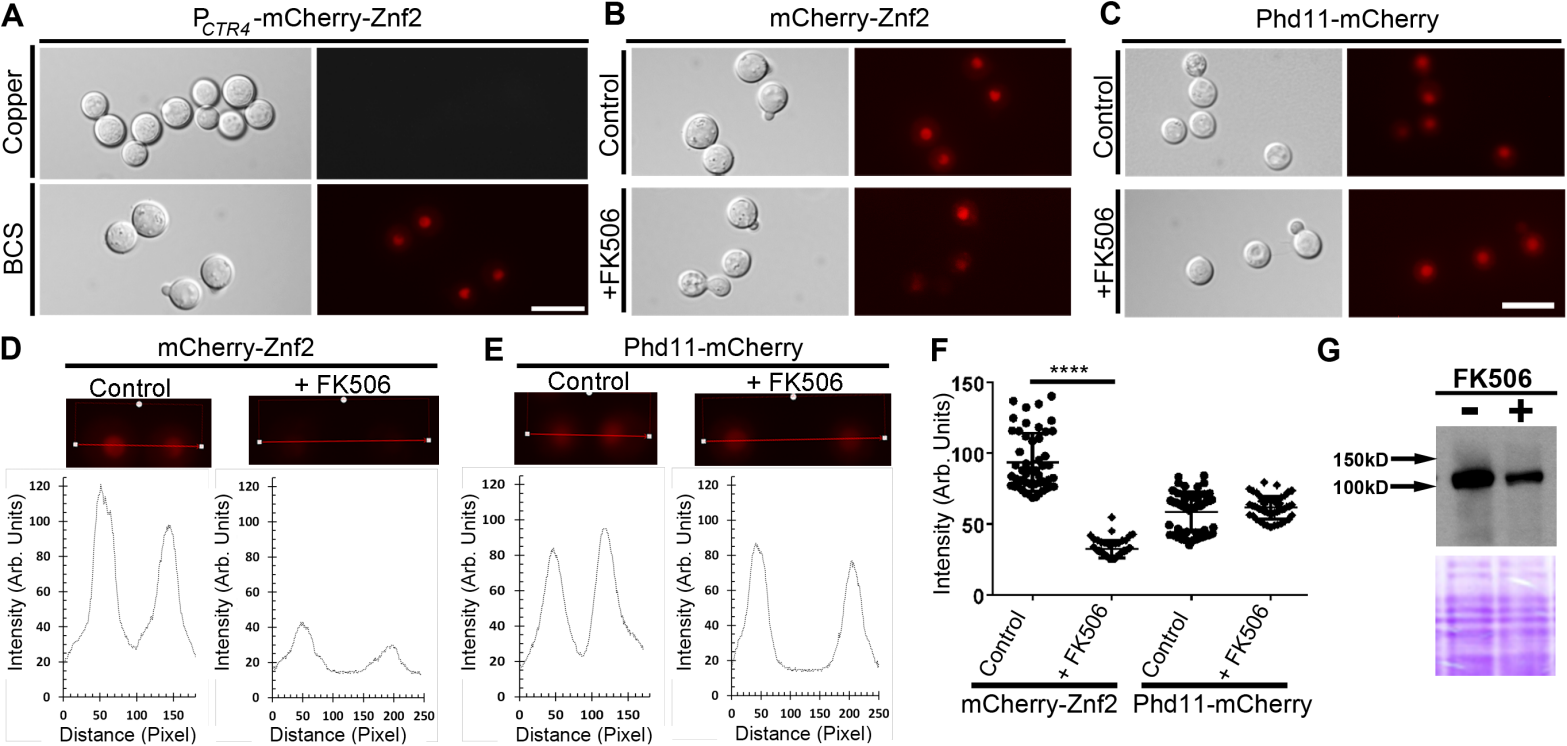
Calcineurin affects the stability of Znf2. **(A)** The P_*CTR4*_-mCherry-Znf2 strain were cultured in YPD medium in the presence of inducer BCS and repressor copper. **(B-C)** Equal number of cells of strains carrying mCherry-Znf2 (B) and Phd11-mCherry (C) were grown either in the presence or the absence of calcineurin inhibitor FK506 for 2 hours after which the images were taken. Relative fluorescence intensity of mCherry-Znf2 **(D)** and Phd11-mCherry **(E)** was plotted along the indicated lines in the images above in the presence or the absence of FK506 treatment. **(F)** Distribution of fluorescence intensity of mCherry-Znf2 and Phd11-mCherry with or without FK506 treatment. ****: p<0.0001. **(G)** Western blot probed for mCherry labelled Znf2 for cells with or without FK506 treatment. Coomassie blue staining of the gel was used as loading control. Scale bars: 10 μm.

To examine if calcineurin affects the localization or the stability of Znf2, we tested the effect of the calcineurin inhibitor FK506 on mCherry-tagged Znf2 that was controlled by the promoter of the copper transporter *CTR4* [51]. The expression of mCherry-Znf2 was suppressed by copper and induced in the presence of the copper chelator BCS (Fig 7A), consistent with our previous studies [57]. When cells expressing mCherry-Znf2 were exposed to FK506, the fluorescence intensity was sharply reduced (Fig 7B, 7D and 7F). However, signals of mCherry-Znf2 could still be detected in the nucleus (Fig 7B), suggesting that treatment with FK506 primarily affected the protein level of Znf2 rather than its nuclear localization. As a control, we used a strain carrying a mCherry tagged Phd11, a protein with two PHD finger domains (plant homeodomains) that are conserved readers of histone modifications in eukaryotes [58]. Treatment with FK506 did not affect either the nuclear localization or the intensity of Phd11 (Fig 7C, 7E and 7F). This suggests that reduced mCherry-Znf2 signal is not a general effect caused by FK506. Reduction in the Znf2 protein level after FK506 treatment was also observed by Western blot analyses (Fig 7E). Collectively, these results suggest that calcineurin may regulate filamentation through controlling the stability of the master regulator Znf2.

## Discussions

The eukaryotic microbe *C*. *neoformans* can undergo bisexual reproduction as well as unisexual reproduction. Unisexual reproduction in this fungus is proposed to have given rise to the sharply skewed population towards the α mating type in the current natural population [9, 11, 15, 16, 59]. However, the inefficiency of unisexual development compared to bisexual development under mating-inducing laboratory conditions challenges this view. One explanation for the conflicting observations is that the laboratory conditions currently used for sexual reproduction might not mimic natural niches of *Cryptococcus* that favor unisexual reproduction. An alternative but not mutually exclusive hypothesis is that there is bifurcation of genetic regulation for unisexual and bisexual development. Certain environmental conditions may favor unisexual development while suppressing the activation of genetic pathways required for bisexual development.

To test this hypothesis, we used the *mat2*Δ mutant, which is blocked for bisexual mating, to search for conditions that specifically promote unisexual development in *Cryptococcus*. We found that *C*. *neoformans* could indeed undergo unisexual development without Mat2. We further showed that filamentation in the *mat2*Δ mutant is independent of pheromone or other components of the pheromone sensing pathway. Furthermore, we found that several disparate conditions (prior heat exposure, copper, or calcium) can induce pheromone-independent filamentation. These conditions are likely encountered by this fungus in nature, as *C*. *neoformans* is known to be associated with soil and vegetation. Some leafy greens such as kale contain copper (~240 μM) and calcium (~37 mM) at concentrations high enough to stimulate unisexual development. Thus, it is conceivable that some natural niches will provide suitable conditions for pheromone-independent unisexual development in *Cryptococcus*.

The pheromone sensing pathway is crucial for bisexual mating in *Cryptococcus* and also in a wide variety of fungal species across different phyla, such as *Saccharomyces*, *Candida*, and *Ustilago* [60–63]. In essence, pheromone triggers non-self-recognition (pheromone produced by α cells preferentially binds to the pheromone receptor present in **a** cells and vice-versa), which then promotes cell fusion. Accordingly, the efficiency of bisexual mating is often quantified by cell fusion events [36]. However, unlike bisexual reproduction, unisexual reproduction can proceed either through cell-cell fusion or endoreplication, and the latter is likely the major route for ploidy increase [3, 9, 16]. Increase in ploidy by endoduplication often occurs as a response to stress in eukaryotes [64–66], and sexual reproduction in many fungi, including *Cryptococcus*, takes place under stressful conditions. After increased ploidy through endoduplication, *Cryptococcus* could complete sexual reproduction by meiosis to return to the haploid state. Contradictory to the non-essentiality of pheromone for unisexual development in *Cryptococcus*, the transcription factor of the pheromone pathway Mat2 was shown to be required for both unisexual and bisexual development under all the mating-inducing conditions tested so far. Thus, this study resolves the conflicting observations for and against the crucial role of the pheromone pathway in unisexual development [36–38, 61]. Collectively, this and previous studies firmly establish that the very process that defines bisexual mating is not crucial for unisexual development.

Some of the important morphological features are conserved in both pheromone-dependent and pheromone-independent sexual development. Both proceed through the formation of hyphae, basidia, and spores. Both require the master regulator of filamentation Znf2 and general stress regulator calcineurin. Filamentation marker Cfl1 is produced by hyphae generated during pheromone-dependent sexual development [31] and during pheromone-independent unisex. The cell surface protein Dha1[40] shows similar localization at basidia produced by pheromone- dependent or independent sexual development. Hence, the key downstream features seem to be conserved in both unisexual and bisexual development.

Our findings demonstrate the possibility of sexual development under conditions and environments where pheromone is not activated, or in strains in which the pheromone pathway is genetically deactivated/non-functional. The observations that the pheromone (*mfα1–3*Δ) and pheromone transporter mutants self-filamented more robustly than the wild type on V8+copper medium, and that the *mat2*Δ mutant showed the extreme phenotypes of being non-filamentous on mating-inducing V8 medium while filamented robustly on V8+copper medium, suggest that the pheromone pathway might even exert an inhibitory effect on unisexual development under certain occasions. Our RNA-seq data suggest that the V8+copper medium is inhibitory to the expression of pheromone and other components of the pheromone pathway. It is possible that Mat2 itself represses genes that promote unisexual development. We are interested in interrogating this hypothesis in the future.

The finding that disparate conditions could trigger pheromone-independent unisexual development reflects the robustness of sexual reproduction in *Cryptococcus*. Likewise, multiple cues such as temperature, nutrition starvation, N-acetyl glucosamine, oxidative stress, and genotoxic agents can induce the commensal and pathogenic fungus *Candida albicans* to switch from the “white” state to the mating competent “opaque” state [67, 68]. Adding to the complexity is that both pheromone-dependent and pheromone-independent sexual reproduction modes likely occur simultaneously in different subpopulations in a wild-type cryptococcal community. The heterogeneity in the sexual mode in the community might reflect or be enforced by the stochastic expression of Mat2 within the population. Consistent with this idea, we noticed that not all cells responded to the calling of pheromone even under a strong mating-inducing condition [40]. Given that *Cryptococcus* is a ubiquitous environmental fungus and an opportunistic pathogen to a wide range of hosts, this fungus might have developed various tactics to propagate sexually under different conditions rather than the risky sole dependence on the pheromone pathway and the rare presence of a compatible mating partner. Hence, sexual reproduction, a well-known evolution feature for adaptation, is itself an adaption to varying selective pressures.

## Methods and materials

### Strains and growth conditions

The strains used in this study are listed in S1 Table. *Cryptococcus* strains were maintained as glycerol stocks at -80°C. Yeast cells were grown on YPD medium (1% yeast extract, 2% Bacto peptone, 2% dextrose, 2% Bacto agar). Mating assays were performed on V8 pH7 solid medium (0.5 g/litre KH_2_PO_4_, 4% Bacto agar, 5% of V8 juice from Campbell Soup Co.) in the dark at 22°C with or without the addition of copper, other metal ions, or the copper chelator BCS as indicated in the texts and figures. Copper at 400 μM was used in most experiments unless indicated otherwise. Other media used for filamentation assays include MS medium (Murashige and Skoog medium minus sucrose, Sigma-Aldrich), YNB medium (6.7 g/L Yeast Nitrogen Base without amino acids, 2% Bacto agar, 2% dextrose), and Filament Agar medium (6.7 g/L Yeast Nitrogen Base without ammonium sulphate, 0.5% glucose, and 4% Bacto-agar at pH 5.0) as described previously [8, 69].

### Gene disruption, complementation, and overexpression

Gene deletion was carried out using the split marker recombination approach as we previously described [70]. Briefly, the 5’ and 3’ sequences of approximately 1 kb that flank the open reading frame of the gene of interest were fused to two third of the NAT or NEO drug resistance marker respectively using overlap PCR. Dominant drug markers NAT and NEO were amplified from the plasmids pPZP-NATcc and pPZP-NEO1 [71]. The gene deletion construct (mixture of two fragments) was introduced into the recipient strain through biolistic transformation as described previously [72]. Gene deletion was confirmed through diagnostic PCR as we described previously [70]. For the mutant strains when bisexual mating was possible, the linkage between the gene deletion and the observed mutant phenotypes was established through genetic linkage assay of micro-dissected basidiospores as described previously [73]. Briefly, meiotic progeny from the cross between a mutant and the congenic wild type of a compatible mating type was dissected. These progeny were analyzed for the mutant phenotype, mating type, and the presence of the gene deletion at the correct genetic locus to establish whether the phenotypes observed are genetically linked to the gene deletion. For complementation of the deletion strains, the ORF and 1–1.5 kb upstream sequence of the gene was amplified using the wild-type genomic DNA as the template. The amplicon was then cloned into the pPZP-NEO1 plasmid. The wild-type allele of the gene together with the drug selection marker was then introduced into the corresponding gene deletion strain through biolistic transformation. To construct overexpression strains, ORFs of the genes of interest were amplified by PCR, digested, and then ligated into the pXL1 plasmid after the *GPD1* promoter region as we described previously [31]. This generates constitutive expression of the gene. For the inducible system, we replaced the P_*GPD1*_ region of the plasmid with the P_*CRT4-2*_ to generate the copper inducible system as described previously [31, 51]. All primers used for generating gene deletion, complementation, or overexpression are listed in S2 Table.

### Generation of mCherry tagged strains

To generate mCherry tagged proteins, the mCheery gene was fused to the C-terminus of *CFL1*, *PHD11*, and *DHA1* as we described previously [31]. To drive the gene expression with their native promoter (e.g. P_*CFL1*_-*CFL1*-mCherry), 1 kb upstream of the gene’s ORF together with its ORF was amplified through PCR and ligated into the pXL1 vector containing mCherry such that mCherry is fused in frame with the C-terminus of the gene [31]. To drive the gene expression with the *GPD1* promoter (e.g. P_*GPD1*_-*DHA1*-mCherry), ORF of *DHA1* fused with mCherry was introduced into pXL1 vector after the P_*GPD1*_ region [40]. The plasmids were then digested and introduced into the *mat2*Δ mutant using biolistic transformation to obtain the respective strains (*mat2*Δ P_*CFL1*_-*CFL1*-mCherry and *mat2*Δ P_*GPD1*_-*DHA1*-mCherry).

### Filamentation and sporulation assays

For self-filamentation, WT and mutant cells of equal optical density measured at 600 nm (OD_600_ = 3) were plated onto V8 or V8+copper medium and incubated in the dark at 22°C for 6 days or as specified in the figures and texts. For most of the phenotypic assays, 400 μM of CuSO_4_ was added to the V8 juice medium. For testing different metal ions, 400 μM of the CaCl_2_, FeCl_3_, and ZnSO_4_ were used. For sporulation and protein localization studies, 150–200 μM of CuSO_4_ was used. Spores were visualized after incubation on V8 medium or V8 medium with copper for 2–3 weeks. For examining the ability of the mutant cells to undergo bisexual mating, non-self-filamentous reference strain of the mating type **a**, JEC20, was used [74].

### Microscopy

Colony images were acquired through a GO21 camera connected to the stereoscope Olympus SZX16 as we described previously [57]. Yeast, hyphae, basidia, and spores were captured by a Zeiss Axiocam 506 camera connected to a Zeiss Imager M2 epifluorescence microscope as we described previously [75]. The filter used for visualizing mCherry was the FL filter set 43 HE cy3 (Carl Zeiss Microscopy).

### RNA extraction, qPCR, and northern blot

RNA extraction and qPCR was performed as we described previously [31]. For extraction of total RNA from cells grown on V8 or V8+copper medium, equal number of cells were plated onto the medium and cells were incubated in the dark at 22°C. For the control samples, cells were grown overnight in liquid YPD. At the indicated time points, cells were collected, washed with cold ddH_2_O, and lyophilized. Total RNA was extracted using Purelink RNA minikit (Life Technologies) followed by DNase treatment (Ambion). The quality and quantity of the RNA samples were analyzed by electrophoresis on a denaturing formaldehyde agarose gel as we described previously [57]. First strand cDNA synthesis was performed using Superscript III cDNA synthesis kit (Life Technology) following the manufacturer’s instructions. Transcript levels were normalized using the constitutively expressed housekeeping gene *TEF1* as we described previously [31]. Primers for real-time PCR are listed in S2 Table.

The procedures for northern blot analyses were the same as we described previously [30]. Briefly, total RNA was extracted from cells grown on V8 or V8+copper medium for 16 hours. Poly (A) tailed RNAs were purified using PolyATtract mRNA isolation System IV (Promega) following manufacturer’s instructions. The random primers DNA labeling System (Life technologies) was used for generating probes for *MF*α and *ACT1*. The primers used are listed in S2 Table.

### RNA-seq analysis

The WT strain XL280 and the *mat2*Δ mutant were cultured on YPD, V8, and V8+copper (400 μM) media. Cells were collected at 16 hours after inoculation for the extraction of total RNA. The total RNA samples were submitted to the TAMU AgriLife Centre for bioinformatics and genomic systems engineering for strand specific RNA-seq following the standard protocol for Illumina Genome Analyzer IIx (http://www.txgen.tamu.edu/?s=sequencing&search.x=0&search.y=0&search=Search) as we described previously [57]. Sequence reads were aligned to the XL280 reference sequence [13] as pairs with Tophat2 [13, 76, 77]. Genes with differential expression was investigated using DESeq [78] and edgeR [79] with default settings. A gene was considered as significantly differentially expressed only when it was identified by both DESeq and edgeR, and passed the false discovery rate cutoff (FDR< = 0.05). IGV software was used for viewing the transcripts [80]. RNA-seq data are deposited at NCBI (BioProject ID: PRJNA344667) as the following SRA files: SRR5272486, SRR5272476, SRR5272474, SRR5272472, SRR5272484, SRR5272482, SRR5272480, and SRR5272478.

### Insertional mutagenesis *via Agrobacterium*-mediated transformation and mutant screen

Insertional mutagenesis was performed in the *mat2*Δ mutant made in the XL280 background (NAT^R^). *Agrobacterium tumefaciens* strain EHA105 containing the Ti-plasmid pPZP-NEO1 was used for the insertional mutagenesis as described previously [30, 71]. *A*. *tumefaciens* was grown overnight in Luria-Bertani medium containing kanamycin at 22°C. Cells were washed twice with sterile water and grown on induction medium containing 100 μM Acetosyringone for additional 6 hours. *Cryptococcus mat2*Δ cells grown overnight in liquid YPD was washed and resuspended in induction medium to obtain the density of 1x10^7^ cells/ml. Equal aliquots of fungal and bacterial cells were mixed and co-cultured on the induction medium (200 μl per drop) for 3 days at 22°C in the dark. The cocultured cells were then collected and plated onto V8+cefotaxime+G418+NAT+copper medium. *Agrobacterium* cells were killed on this selective medium (antibiotic cefotaxime) and transformants with T-DNA (confer G418 resistance) would be able to survive. Most colonies were able to filament on the V8+copper medium, similar to the *mat2*Δ parental strain. Mutants that only grew in the yeast form after 2 weeks of incubation were selected. To identify insertion sites through inverse PCR coupled with sequencing, genomic DNA from the selected mutants was digested with a restriction enzyme, purified, and self-ligated as we described previously [30]. Primers AI076/Ai077 were used for inverse PCR and sequencing as we described previously [30, 71]. After sequencing, flanking region sequences were used for BLAST search against *C*. *neoformans* serotype D genome database at Genebank to identify the insertion sites and the genetic loci affected by the insertion.

### Genomic sequencing and identification of the insertional sites

A total of 47 candidate insertional mutants which could not filament on V8+copper medium were selected. These candidate strains were grown overnight in YPD liquid medium. Genomic DNA was extracted following cetyltrimethylammonium bromide (CTAB) extraction protocol as previously described [81, 82]. Genomic DNA from 47 individual selected mutants was pooled into four groups, with each group consisting DNA from 11–12 mutants. 10 μg of total DNA from each pool was submitted to the TAMU AgriLife, Centre for bioinformatics and genomic systems engineering for sequencing (Illumina Miseq 175 bp x 175 bp, paired end reads). Each group yielded 1.1x10^8^ to 1.75x10^8^ reads pairs (129.5 M pairs), yielding an average 12x coverage of *Cryptococcus* genome per strain. Analysis of the insertion sites was performed using the AIMHII approach as recently described for *Cryptococcus* T-DNA insertional mutants [43].

### Effect of calcineurin inhibitor on the intensity and localization mCherry-Znf2

Strains with mCherry tagged Znf2 or Phd11 (control) were grown overnight in inducing condition in liquid YPD culture for 10 hours and cells were examined under a Zeiss Imager M2 epifluorescence microscope to visualize the mCherry signal. Cells of equal density (OD_600_ = 3) were then treated with or without FK506 (2 μg/ml) for additional 2 hours. Cells were then briefly washed with ddH_2_O and their images were taken with Zeiss Imager M2 epifluorescence microscope using same exposure time for each sample. Data for intensity plot was obtained through ZEN image software (Carl Zeiss Microscopy, NY).

### Protein extraction and Western blot

Protein extraction and western blotting was performed as described previously [83–85]. P_*CTR4*_ –mCherry-Znf2 strains were grown in liquid YPD+BCS for 10 hours. Half of the samples were grown for 2 additional hours with the addition of FK506 (2 μg/ml) whereas the other half of the samples were grown without FK506. The cells were lyophilized after washing with cold phosphate-buffered saline (PBS). The dried cells mixed with glass beads were disrupted by a Cell Disrupter (Next Advance). Total protein extraction was carried out using the lysis buffer (300 mM NaCl, 2mM EDTA, 25mM HEPES (pH 7.5), proteinase inhibitor). The sample was denatured with SDS containing loading buffer. Samples were separated on SDS -12% PAGE gel and were transferred to a membrane using TE 70 ECL semidry transfer unit (GE healthcare) for 1 hour at 30V. Anti-mCherry primary antibody (1/2000 dilution) and rabbit anti-mouse secondary antibody (1/10,000 dilution) were used to detect mCherry signals. Signal detection was carried out using chemiluminescence (ECL) system according to the manufacturer’s instruction (Pierce).

## Supporting information

S1 Fig Copper trigged Mat2-independent filamentation is not limited to V8 juice medium. Wild-type strains XL280α and XL280**a** and the corresponding *mat2*Δ mutant strains were cultured on V8 juice medium **(A)**, Filamentation Agar (FA) medium **(B)**, or Yeast Nitrogen Base (YNB) medium **(C)** with or without Cu^2+^ (400 μM) at 22°C for one week. Images of the whole colonies (upper panel) and the edges of the colonies (lower panel) are shown. The scale bar for images of the whole colonies is 1 mm and for images of the colony edges is 500 μm.
(PDF)

S2 Fig The *mat2*Δ mutant in JEC21 background can also filament on V8+copper medium. JEC21 filamented poorly on V8 medium and this strain filamented better on V8+Cu^2+^ medium (top panel). The *mat2*Δ strain in JEC21 background failed to filament on V8 medium, but it produced robust filamentation on V8+Cu^2+^ medium (bottom panel).
(PDF)

S3 Fig The *mat2*Δ mutant has the ability to form basidium and spores. The *mat2*Δ mutant was grown on V8 supplemented with 150 μM of copper for 2–3 weeks. The *mat2*Δ mutant filamented and produced basidia and spores, albeit at a reduced level compared to wild type cultured on V8 medium. Scale bars: 5 μm.
(PDF)

S4 Fig The *mat2*Δ mutants made in the congenic pair strains JEC21α/JEC20a filament on copper media. Wild-type strains JEC21α, JEC20**a**, and the corresponding *mat2*Δ mutants were cultured on V8 or V8+400 μM Cu^2+^ medium (**A**), or FA or FA+200 μM Cu^2+^ medium **(B)** at 22°C for 7 days. Images of the whole colonies (upper panel) and the colony edges (lower panel) are shown. The scale bar for images of whole colonies is 1 mm and the scale bar for images of colony edges is 500 μm.
(PDF)

S5 Fig The *znf2*Δ*mat2*Δ mutant cannot mate with a wild-type partner of the opposite mating type. JEC20**a** cultured alone did not produce filaments. The cross between the *znf2*Δ α mutant with JEC20**a** produced filaments. The cross between the *znf2*Δ*mat2*Δ α mutant with JEC20**a** failed to produce any filaments. Scale bar: 200 μm.
(PDF)

S6 Fig Deletion of all three pheromone genes *MFα1*,*2*,*3*, or the pheromone transporter gene *STE6* did not abolish filamentation on V8+Cu^2+^ medium. Cells were cultured on V8 or V8+copper medium. Scale bar: 500 μm.
(PDF)

S7 Fig Insertional mutagenesis screen. **(A)** Insertional mutagenesis was performed in the *mat2*Δ mutant background. The parental *mat2*Δ mutant produced robust filamentation on V8+copper medium. One representative insertional mutant that failed to produce any filaments on V8+copper medium was shown. **(B)** Chromosomal distribution of the insertional sites. Red lines represent insertions within the ORF of the genes and green lines indicate insertions in the intergenic regions. Bold line indicates more than one insertion within that region.
(PDF)

S8 Fig **(A)** Venn diagram showing the shared and unique genes between the *mat2*Δ mutant and WT cells grown on V8+copper compared to those grown on V8. **(B)** Transcriptome analysis of the RNA-seq data showed the genes of the calcineurin pathway that were upregulated more than 3 fold in the *mat2*Δ mutant. **(C)** The *mat2*Δ mutant can filament in response to CaCl_2_. Cells were grown on 1500 μM CaCl_2_ for 3 weeks before the images were taken. Scale bar: 200 μm**. (D)** Deletion of *CRZ1* did not affect pheromone independent filamentation. WT, *mat2*Δ, *crz1*Δ, and *crz1*Δ *mat2*Δ strains were cultured on V8 or V8+copper medium for 6 days. WT and the *crz1*Δ mutant filamented on both V8 and V8+copper medium. The *mat2*Δ mutant and the *crz1*Δ*mat2*Δ double mutant did not filament on V8 medium, but they produced robust filamentation on V8+copper medium. Scale bar: 500 μm.
(PDF)

S9 Fig Test for temperature sensitivity. WT, *cna1*Δ, *cnb1*Δ, *crz1*Δ, *cna1*Δ m*at2*Δ, *cnb1*Δ m*at2*Δ, and *crz1*Δ m*at2*Δ strains were cultured on YPD medium at 22°C or at 37°C.
(PDF)

S1 Table Strains used in this study. (DOCX)

S2 Table Primers used in this study. (DOCX)

S3 Table Genetic loci affected by the insertion sites. (XLSX)

S4 Table Genes upregulated by more than 2 fold in *mat2*Δ mutant in V8+copper compared to *mat2*Δ mutant grown on V8. (XLSX)
