# Supplementary figures and images for: Pheromone independent unisexual development in Cryptococcus neoformans
Source: PLoS Genet. 2017 May 3;13(5):e1006772. doi: 10.1371/journal.pgen.1006772 (PMC5435349; doi:10.1371/journal.pgen.1006772)

Figure S1

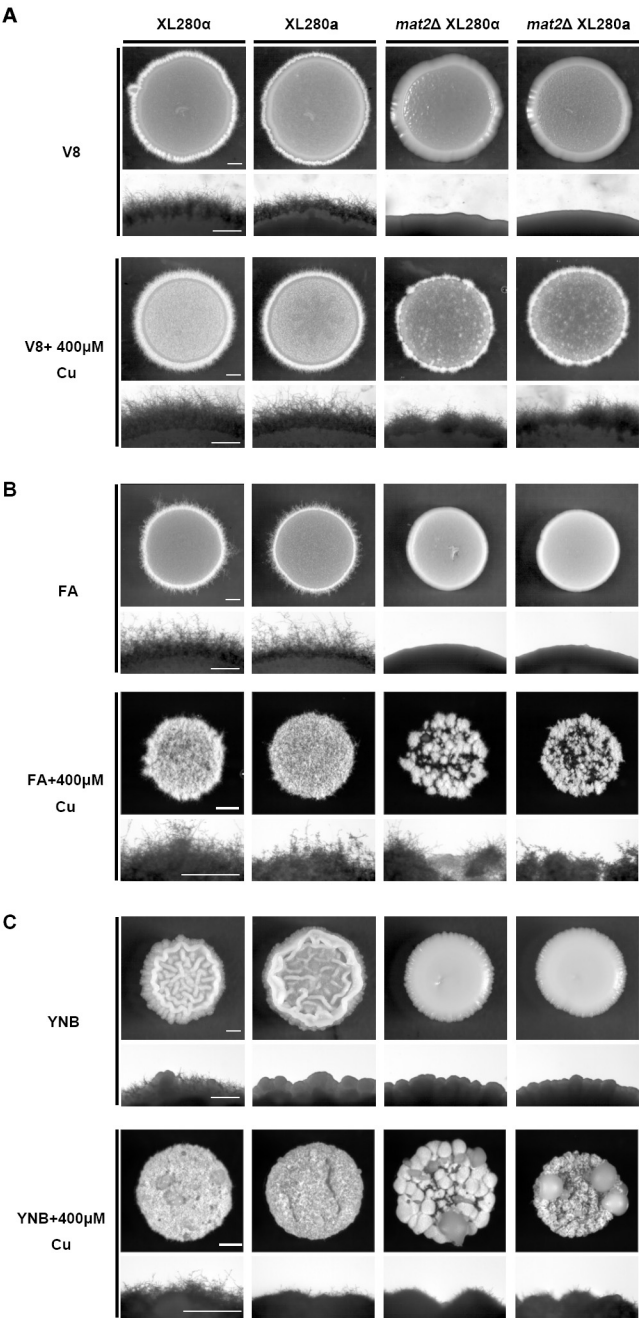

Supplement: S1 Fig — Wild-type strains XL280α and XL280a and the corresponding mat2Δ mutant strains were cultured on V8 juice medium (A), Filamentation Agar (FA) medium (B), or Yeast Nitrogen Base (YNB) medium (C) with or without Cu2+ (400 μM) at 22°C for one week. Images of the whole colonies (upper panel) and the edges of the colonies (lower panel) are shown. The scale bar for images of the whole colonies is 1 mm and for images of the colony edges is 500 μm. (PDF) [file pgen.1006772.s001.pdf]

Figure S2

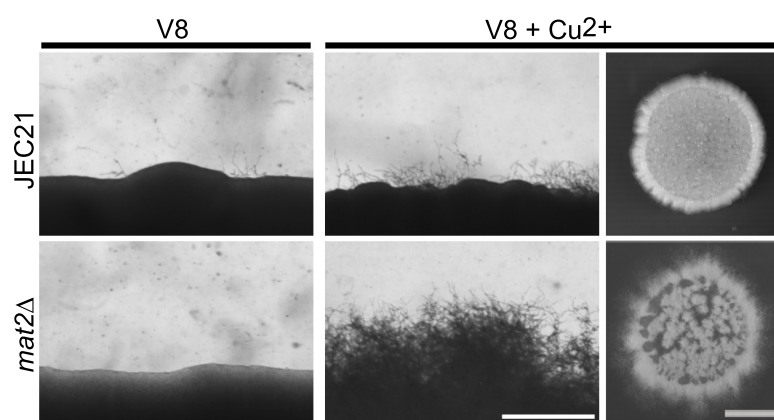

Supplement: S2 Fig — JEC21 filamented poorly on V8 medium and this strain filamented better on V8+Cu2+ medium (top panel). The mat2Δ strain in JEC21 background failed to filament on V8 medium, but it produced robust filamentation on V8+Cu2+ medium (bottom panel). (PDF) [file pgen.1006772.s002.pdf]

Figure S3

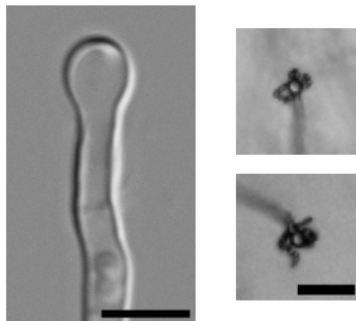

Supplement: S3 Fig — The mat2Δ mutant was grown on V8 supplemented with 150 μM of copper for 2–3 weeks. The mat2Δ mutant filamented and produced basidia and spores, albeit at a reduced level compared to wild type cultured on V8 medium. Scale bars: 5 μm. (PDF) [file pgen.1006772.s003.pdf]

Figure S4

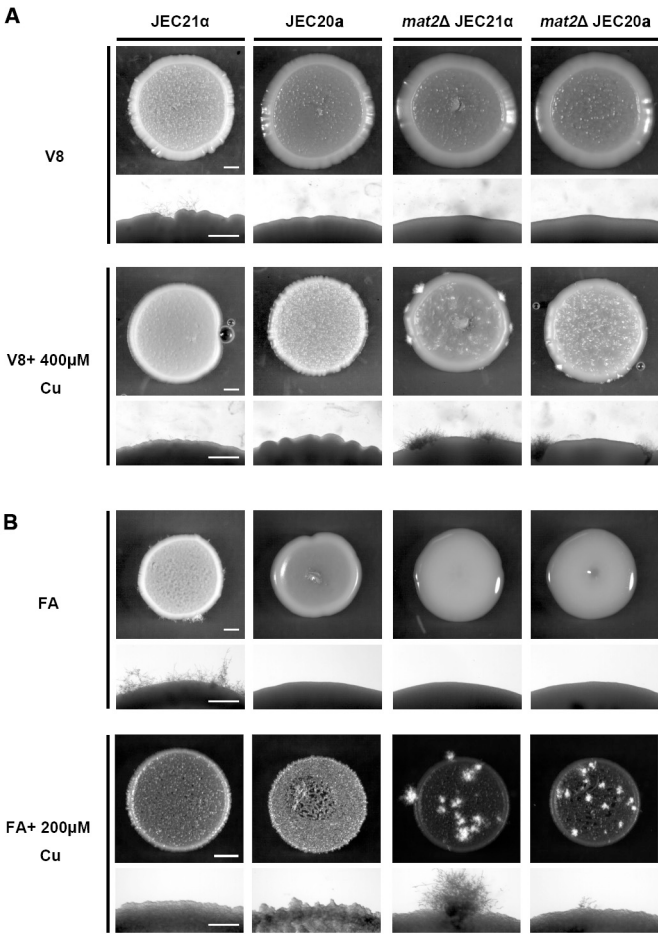

Supplement: S4 Fig — Wild-type strains JEC21α, JEC20a, and the corresponding mat2Δ mutants were cultured on V8 or V8+400 μM Cu2+ medium (A), or FA or FA+200 μM Cu2+ medium (B) at 22°C for 7 days. Images of the whole colonies (upper panel) and the colony edges (lower panel) are shown. The scale bar for images of whole colonies is 1 mm and the scale bar for images of colony edges is 500 μm. (PDF) [file pgen.1006772.s004.pdf]

Figure S5

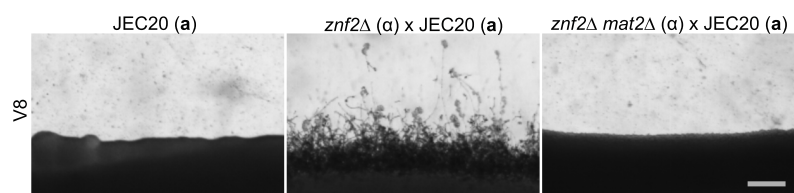

Supplement: S5 Fig — JEC20a cultured alone did not produce filaments. The cross between the znf2Δ α mutant with JEC20a produced filaments. The cross between the znf2Δmat2Δ α mutant with JEC20a failed to produce any filaments. Scale bar: 200 μm. (PDF) [file pgen.1006772.s005.pdf]

Figure S6

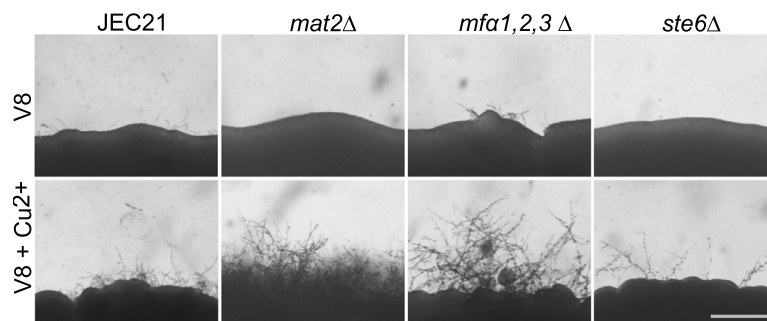

Supplement: S6 Fig — Cells were cultured on V8 or V8+copper medium. Scale bar: 500 μm. (PDF) [file pgen.1006772.s006.pdf]

Figure S7

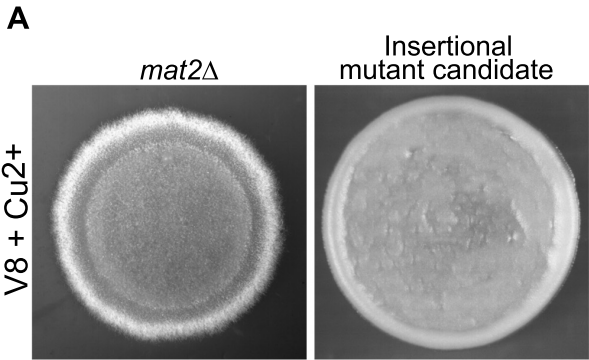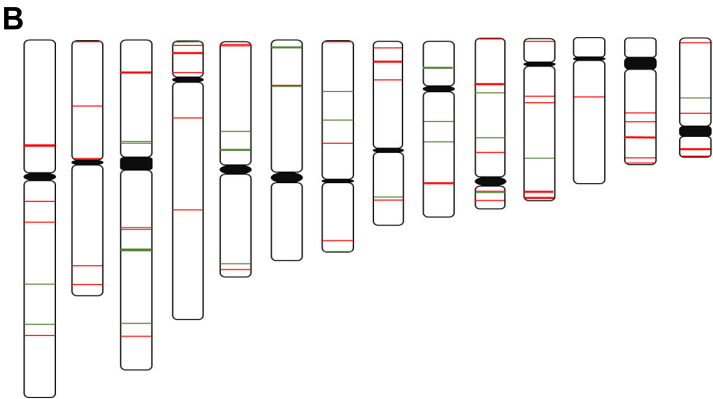

Supplement: S7 Fig — (A) Insertional mutagenesis was performed in the mat2Δ mutant background. The parental mat2Δ mutant produced robust filamentation on V8+copper medium. One representative insertional mutant that failed to produce any filaments on V8+copper medium was shown. (B) Chromosomal distribution of the insertional sites. Red lines represent insertions within the ORF of the genes and green lines indicate insertions in the intergenic regions. Bold line indicates more than one insertion within that region. (PDF) [file pgen.1006772.s007.pdf]

Figure S8

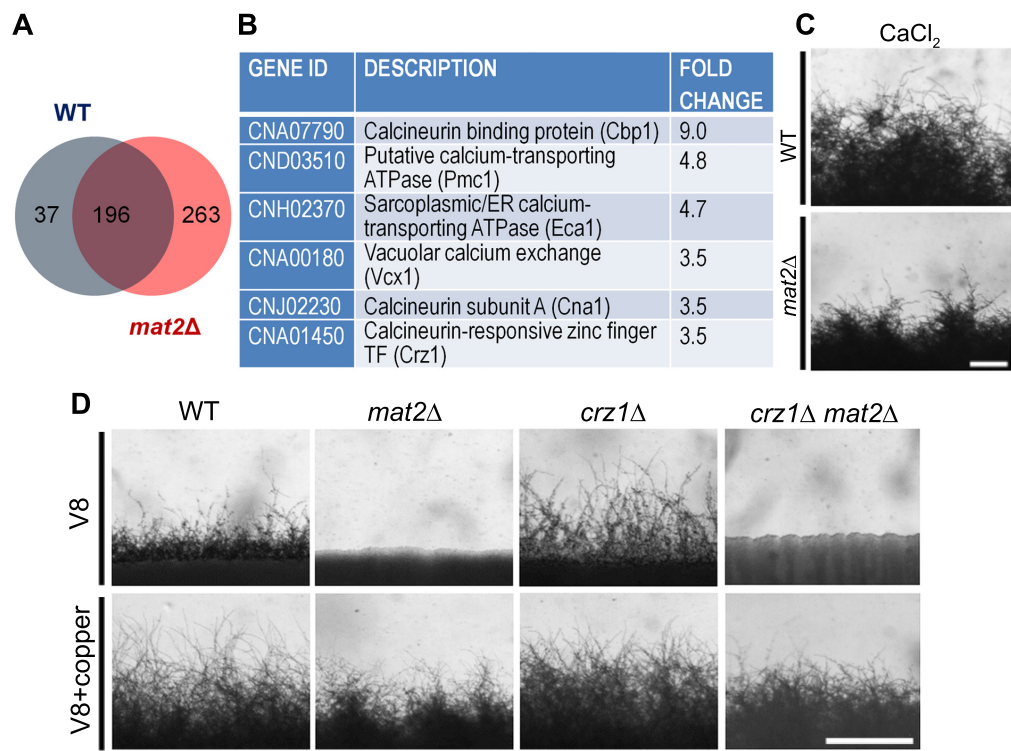

Supplement: S8 Fig — (A) Venn diagram showing the shared and unique genes between the mat2Δ mutant and WT cells grown on V8+copper compared to those grown on V8. (B) Transcriptome analysis of the RNA-seq data showed the genes of the calcineurin pathway that were upregulated more than 3 fold in the mat2Δ mutant. (C) The mat2Δ mutant can filament in response to CaCl2. Cells were grown on 1500 μM CaCl2 for 3 weeks before the images were taken. Scale bar: 200 μm. (D) Deletion of CRZ1 did not affect pheromone independent filamentation. WT, mat2Δ, crz1Δ, and crz1Δ mat2Δ strains were cultured on V8 or V8+copper medium for 6 days. WT and the crz1Δ mutant filamented on both V8 and V8+copper medium. The mat2Δ mutant and the crz1Δmat2Δ double mutant did not filament on V8 medium, but they produced robust filamentation on V8+copper medium. Scale bar: 500 μm. (PDF) [file pgen.1006772.s008.pdf]

Figure S9

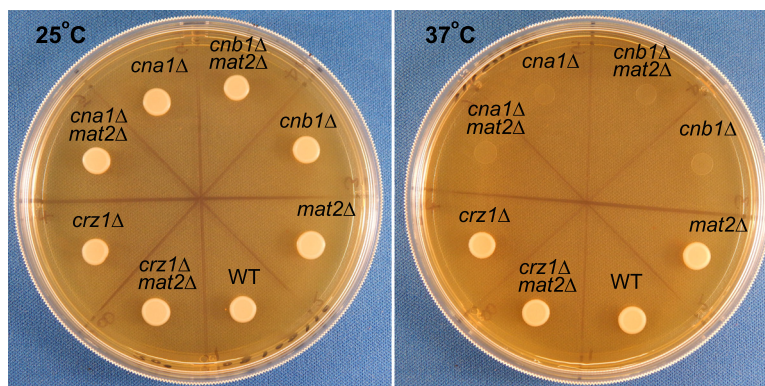

Supplement: S9 Fig — WT, cna1Δ, cnb1Δ, crz1Δ, cna1Δ mat2Δ, cnb1Δ mat2Δ, and crz1Δ mat2Δ strains were cultured on YPD medium at 22°C or at 37°C. (PDF) [file pgen.1006772.s009.pdf]
