# Supplementary material for: Pheromone independent unisexual development in Cryptococcus neoformans
Source: PLoS Genet. 2017 May 3;13(5):e1006772. doi: 10.1371/journal.pgen.1006772 (PMC5435349; doi:10.1371/journal.pgen.1006772)
Supplement: S1 Table — (DOCX) [file pgen.1006772.s010.docx]

Supplemental Table 1. Strains used in the study

| Strain name | Genotype | Source and comments | Background |
| --- | --- | --- | --- |
| XL280α | *MAT*α (WT) | [1] | XL280 |
| XL280**a** | *MAT***a** (WT) | Congenic with XL280α [2] | XL280 |
| LW192α | P*_CFL1_-CFL1::mCherry:: NEO^r^* | [3] | XL280 |
| JEC21α | *MAT*α (WT) | [4, 5] | JEC21 |
| JEC20**a** | *MAT***a** (WT) | Congenic with JEC21 [4, 5] |  |
| KN99α | wild type | Isogenic with H99 [6] | H99 |
| KN99**a** | wild type | Congenic with H99/KN99α [6] | H99 |
| XL574α | *znf2*::*NAT^r^* | [7] | XL280 |
| XL942α | *mat2::NAT^r^* | [8] | XL280 |
| RG335**a** | *mat2::NEO^r^* | This study | XL280 |
| RG354α | *cnb1::NEO^r^* | This study | XL280 |
| RG359α | *cnb1::NEO^r^mat2::NAT^r^* | This study | XL280 |
| RG426α | *cna1::NAT^r^* | This study | XL280 |
| RG437α | *cna1::NAT^r^ mat2::HYG^r^* | This study | XL280 |
| RG438α | P­*_CTR4_*-*mCherry-ZNF2-NEO^r^cna1::NAT* | This study | XL280 |
| RG427α | *cna1::NAT^r^CNA1::NEO* | This study | XL280 |
| RG428α | *cna1::NAT^r^CNA1::NEO* | This study | XL280 |
| XL926α | *mat2::NAT^r^* | [8] | JEC21 |
| XL961**a** | *mat2::NEO^r^* | [8] | JEC21 |
| LW329α | *mat2::NAT^r^* P*_CFL1_-CFL1::mCherry:: NEO^r^* | This study | XL280 |
| RG446α | *mat2::NAT^r^* P*_GPD1_-DHA1::mCherry:: NEO^r^* | This study | XL280 |
| RG421α | P*_GPD1_-DHA1::mCherry:: NEO^r^* | This study | XL280 |
| RG410α | *cnb1::NEO^r^mat2::NAT^r^P_GPD1_-ZNF2::HYG^r^* | This study | XL280 |
| RG341α | *mat2::NAT^r^MAT2::NEO^r^* | This study | XL280 |
| RG450α | *mat2::NAT^r^P_GPD1_-ZNF2::HYG^r^* | This study | XL280 |
| RG349α | *crz1*::*NEO* | This study | XL280 |
| RG379α | *crz1*::*NEO*, *mat2*::*NAT* | This study | XL280 |
| RG318α | *znf2*::*NAT* *mat2*::*NEO* | This study | XL280 |
| LW538α | P­*_CTR4_*-*mCherry-ZNF2-NEO^r^* | This study | XL280 |
| WSC18α | *mfα1::ADE2 mfα2,3::URA5* *ade2 ura5* | [9] | JEC21 |
| YPH134α | *ste6::URA5* | [10] | JEC21 |
| YM72α | P*_GPD1_*-*PHD11*-mCherry::NEO | This study | XL280 |

1. Lin X, Huang JC, Mitchell TG, Heitman J. Virulence Attributes and Hyphal Growth of *C. neoformans* Are Quantitative Traits and the *MAT*α Allele Enhances Filamentation. PLoS Genet. 2006;2(11):e187. doi: 10.1371/journal.pgen.0020187.

2. Zhai B, Zhu P, Foyle D, Upadhyay S, Idnurm A, Lin X. Congenic Strains of the Filamentous Form of *Cryptococcus neoformans* for Studies of Fungal Morphogenesis and Virulence. Infection and Immunity. 2013;81(7):2626-37. doi: 10.1128/iai.00259-13.

3. Wang L, Zhai B, Lin X. The link between morphotype transition and virulence in *Cryptococcus neoformans*. PLoS pathogens. 2012;8(6):e1002765. Epub 2012/06/28. doi: 10.1371/journal.ppat.1002765. PubMed PMID: 22737071; PubMed Central PMCID: PMC3380952.

4. Kwon-Chung KJ, Kozel TR, Edman JC, Polacheck I, Ellis D, Shinoda T, et al. Recent advances in biology and immunology of *Cryptococcus neoformans*. J Med Vet Mycol. 1992;30 Suppl 1:133-42. PubMed PMID: 1474438.

5. Heitman J, Allen B, Alspaugh JA, Kwon-Chung KJ. On the origins of congenic *MAT*a and *MAT***a** strains of the pathogenic yeast *Cryptococcus neoformans*. Fungal Genet Biol. 1999;28(1):1-5. PubMed PMID: 10512666.

6. Nielsen K, Cox GM, Wang P, Toffaletti DL, Perfect JR, Heitman J. Sexual cycle of *Cryptococcus neoformans* var. *grubii* and virulence of congenic **a** and alpha isolates. Infection and immunity. 2003;71(9):4831-41. PubMed PMID: 12933823.

7. Lin X, Jackson JC, Feretzaki M, Xue C, Heitman J. Transcription factors Mat2 and Znf2 operate cellular circuits orchestrating opposite and same-sex mating in *Cryptococcus neoformans*. PLoS genetics. 2010;6(5):e1000953.

8. Lin X, Jackson JC, Feretzaki M, Xue C, Heitman J. Transcription Factors Mat2 and Znf2 Operate Cellular Circuits Orchestrating Opposite- and Same-Sex Mating in *Cryptococcus neoformans*. PLoS Genet. 2010;6(5):e1000953. doi: 10.1371/journal.pgen.1000953.

9. Shen W-C, Davidson RC, Cox GM, Heitman J. Pheromones Stimulate Mating and Differentiation via Paracrine and Autocrine Signaling in Cryptococcus neoformans. Eukaryotic Cell. 2002;1(3):366-77. doi: 10.1128/EC.1.3.366-377.2002. PubMed PMID: PMC118021.

10. Hsueh Y-P, Shen W-C. A Homolog of Ste6, the a-Factor Transporter in Saccharomyces cerevisiae, Is Required for Mating but Not for Monokaryotic Fruiting in Cryptococcus neoformans. Eukaryotic Cell. 2005;4(1):147-55. doi: 10.1128/EC.4.1.147-155.2005. PubMed PMID: PMC544149.
