# Supplementary material for: Pheromone independent unisexual development in Cryptococcus neoformans
Source: PLoS Genet. 2017 May 3;13(5):e1006772. doi: 10.1371/journal.pgen.1006772 (PMC5435349; doi:10.1371/journal.pgen.1006772)
Supplement: S2 Table — (DOCX) [file pgen.1006772.s011.docx]

| Supplemental Table 2: Primers used in this study | |  |
| --- | --- | --- |
| **Primer name** | **Primer number** | **Primer sequence** |
| *CNA1* gene deletion | Linlab3732 | ATGTGCCTCAGCCTATCTC |
|  | Linlab3733 | CTGGCCGTCGTTTTACCGGAAGTTGACTGTTTGGT |
|  | Linlab3734 | GTCATAGCTGTTTCCTGGTTTCGAACGATGGAGTT |
|  | Linlab3735 | CGAGTCTGAGTTGATTGAGA |
|  | Linlab3736 | GAACCTTCCACCGAATTAG |
|  | Linlab3737 | ACTCAGACCGCGAATGC |
|  | Linlab3738 | CACATCTCGGATCAAGTGC |
|  | Linlab3739 | GAAACCCAACAACAATGATG |
| *CNB1* gene deletion | Linlab3740 | ATTACTTTGATGGTGGTCAC |
|  | Linlab3741 | GATCTTGCCACTCTTGAGG |
|  | Linlab3742 | CTGGCCGTCGTTTTACGATGGATCTGATGATGAAAC |
|  | Linlab3743 | GTCATAGCTGTTTCCTGAGTATGTCTTTCTCAAAATCG |
|  | Linlab3744 | GATGGCTCTTCACCTCATA |
|  | Linlab3745 | GATACAAAACACCTGATGGT |
|  | Linlab3746 | ATCCTCCATGTTCAACTCTC |
|  | Linlab3747 | CCTAATGGAGATTCAATCAG |
| *CNA1* complementation | Linlab3935 | GATTTCGTTGACGGATTTGGTC |
|  | Linlab3936 | GATTCTCCCAATCTGCTGGAT |
|  | Linlab3937 | TGCAAGCTTCGGCGATTTGA |
|  | Linlab3938 | CGCTTCTTTAATACCTTCG |
|  | Linlab3939 | GAAACCAGCATCTACTCTCACGCTTCTTTAATACCTTCG |
|  | Linlab3940 | TGAGAGTAGATGCTGGTTTC |
|  | Linlab3941 | CGAAGGTATTAAAGAAGCGTGAGAGTAGATGCTGGTTTC |
|  | Linlab3942 | ATAGGAACTTTCTCTCAGCG |
|  | Linlab3943 | GCTCTTGCTAGGTGCTATC |
|  | Linlab3944 | CTGGCCGTCGTTTTACGCTCTTGCTAGGTGCTATC |
|  | Linlab3946 | AGCGCAACGCAATTAATGTGA |
| *CRZ1* gene deletion | Linlab 3764 | CTATGCTAGGTGGGAGATTT |
|  | Linlab 3765 | ATGCATAATTATGTACCCGA |
|  | Linlab 3766 | CTGGCCGTCGTTTTACATTATAGGGGCGACTGAT |
|  | Linlab 3767 | GTCATAGCTGTTTCCTGGACGTACAAGGGCATCAA |
|  | Linlab 3768 | CATGTTACTTTACGAGGTGAG |
|  | Linlab 3769 | GTCGAGGTGCAGTGATTC |
|  | Linlab 3770 | CAACACCAGCAGTTCAACT |
|  | Linlab 3771 | CACATTCAAATAGTCGCAAA |
|  | Linlab 3772 | GATCGAGTCTAGGCAACTGT |
| *MAT2* complementation | Linlab 3284 | ATTCGAT GCGGCCGCACTCGTAATCACGTTTTCG |
|  | Linlab3470 | GATTCACGCGATCGCGGCGTCTAAGCCTGAAG |
| *Mfalpha1* RTPCR | Linlab1267 | ATCTTCACCACCTTCACTTCT |
|  | Linlab1268 | CTAGGCGATGACACAAAGG |
| *CFL1* RTPCR | Linlab795 | GGTCTCTCCATGCTTGTACC |
|  | Linlab796 | CCAGATTTGCAGCTGTAGAC |
| *MAT2* RTPCR | Linlab 975 | GCTCCTCGCTACATCTCCTCA |
|  | Linlab 976 | TGTTTCGGTCTACGATACCAGTT |
| *TEF1* RTPCR | Linlab329 | CGTCACCACTGAAGTCAAGT |
|  | Linlab330 | AGAAGCAGCCTCCATAGG |
| DHA1 for mCherry plasmid | Linlab1966 | CCGGCCGGCCATGTTCTCGTCCACTATTGCG |
|  | Linlab1798 | GATTCAC*GCGATCGC*CAGCTGGAGAGTGACAGA |
